# Circular RNA circ‐TNPO3 inhibits clear cell renal cell carcinoma metastasis by binding to IGF2BP2 and destabilizing SERPINH1 mRNA

**DOI:** 10.1002/ctm2.994

**Published:** 2022-07-25

**Authors:** Xiaojuan Pan, Bo Huang, Qiang Ma, Junwu Ren, Yuying Liu, Cong Wang, Dawei Zhang, Jian Fu, Lingyu Ran, Ting Yu, Haiping Li, Xiaolin Wang, Feifei Yang, Ce Liang, Yuying Zhang, Shimin Wang, Jingjing Ren, Wei Li, Yongquan Wang, Bin Xiao

**Affiliations:** ^1^ College of Pharmacy Chongqing Medical University Chongqing P. R. China; ^2^ Department of Urology Southwest Hospital Army Medical University Chongqing P. R. China; ^3^ Department of Kidney Southwest Hospital Army Medical University Chongqing P. R. China; ^4^ Department of Clinical Laboratory The 89th Hospital of The People's Liberation Army Weifang P. R. China; ^5^ Department of Pharmacy Southwest Hospital Army Medical University Chongqing P. R. China

**Keywords:** circ‐TNPO3, clear cell renal cell carcinoma, IGF2BP2, SERPINH1

## Abstract

**Background:**

Clear cell renal cell carcinoma (ccRCC) is a common malignant tumour of the urinary tract. The major causes of poor prognosis are the lack of early diagnosis and metastasis. Accumulating research reveals that circular RNAs (circRNAs) can play key roles in the development and the progression of cancer. However, the role of circRNAs in ccRCC is still uncertain.

**Methods:**

The circRNAs microarray (*n* = 4) was performed to investigate the circRNAs with differential expression in ccRCC tissues. The candidate circRNA was selected based on the cut‐off criteria, such as circRNA expression abundance, circRNA size and the design of divergent primers. The circ‐transportin‐3 (TNPO3) levels in ccRCC tissues were tested by quantitative real‐time (qRT)‐PCR (*n* = 110). The characteristics and subcellular localization of circ‐TNPO3 were identified via RNase R assay, qRT‐PCR and fluorescence in situ hybridization (FISH). Then, we explored the biological roles of circ‐TNPO3 in ccRCC via the function experiments in vitro and in vivo. RNA pull‐down, RNA immunoprecipitation, bioinformatic analysis, RNA‐FISH assays and rescue assays were applied to validate the interactions between circ‐TNPO3, insulin‐like growth factor 2 mRNA‐binding protein 2 (IGF2BP2) and serpin family H member 1 (SERPINH1) to uncover the underlying molecular mechanisms of circ‐TNPO3.

**Results:**

We detected the obvious downregulation of circ‐TNPO3 in ccRCC compared to matched adjacent normal tissues (*n* = 110). The lower circ‐TNPO3 expression was found in ccRCC patients with distant metastasis, higher World Health Organization/International Society of Urologic Pathologists (WHO/ISUP) grade and more advanced tumour T stage. In vitro and in vivo, circ‐TNPO3 significantly suppressed the proliferation and migration of ccRCC cells. Mechanistically, we elucidated that circ‐TNPO3 directly bound to IGF2BP2 protein and then destabilized SERPINH1 mRNA. Moreover, IGF2BP2/SERPINH1 axis was responsible for circ‐TNPO3's function of inhibiting ccRCC metastasis. Epithelial splicing regulatory protein 1 (ESRP1) was probably involved in the biogenesis of circ‐TNPO3.

**Conclusions:**

Circ‐TNPO3 can suppress ccRCC progression and metastasis via directly binding to IGF2BP2 protein and destabilizing SERPINH1 mRNA. Circ‐TNPO3 may act as a potential target for ccRCC treatment.

## Graphical abstract

- Circ‐TNPO3 is significantly downregulated in ccRCC.
- Circ‐TNPO3 can suppress ccRCC metastasis by directly binding to IGF2BP2 protein and destabilizing SERPINH1 mRNA, in turn, regulating the SERPINH1‐SNAIL/SLUG axis.
- ESRP1 is involved in the biogenesis of circ‐TNPO3 by targeting the flanking intron.

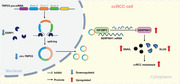

## 1 BACKGROUND

Renal cell carcinoma (RCC) has the second highest mortality rate of all urologic tumours, and its global incidence is steadily rising.^1^ In 2020, globally, 179 368 deaths and 431 288 new cases due to RCC were reported.^2^ Clear cell RCC (ccRCC) is one of the most common subtypes of RCC, accounting for nearly 70%–75% of all cases.^3^, ^4^ The mainstay treatment for localized ccRCC is surgical resection. Nevertheless, ∼30% of patients with localized ccRCC ultimately develop recurrence and metastases, resulting in high mortality.^5^, ^6^ Although considerable improvements in the diagnosis and 5‐year survival rate have been made, the overall prognosis is far from satisfactory.^7^, ^8^ Therefore, it is crucial to deeply evaluate the molecular mechanisms of pathogenesis and metastases and to identify novel reliable therapeutic targets for ccRCC.

A newly discovered category of non‐coding RNAs, known as circular RNAs (circRNAs), have a covalent closed‐loop structure and absence of 5′ caps and 3′ ends.^9^, ^10^ Existing studies have shown that circRNAs have diverse functions in the regulation of gene expression, such as miRNA sponges, alternative splicing, RNA–protein interactions as well as translation ability.^11^, ^12^, ^13^ Further, circRNAs can participate in the development and progression of many cancer types, such as breast cancer,^14^ lung adenocarcinoma,^15^ hepatocellular carcinoma,^16^ prostate cancer^17^ and RCC.^18^ Thus, a comprehensive understanding of circRNAs in ccRCC is warranted.

The interaction between circRNAs and RNA‐binding protein is widely reported to play a critical role during tumour progression.^19^ Insulin‐like growth factor 2 mRNA‐binding protein 2 (IGF2BP2, also named as IMP2) is an important RNA‐binding protein that modulates the translation, localization and stability of RNA and plays a critical role in the post‐transcriptional regulation of RNA.^20^ IGF2BP2 is reportedly dysregulated in many cancers^21^; however, its role in ccRCC is unclear. The serpin family H member 1 (SERPINH1), also called HSP47, is a molecular chaperon necessary in the biosynthetic pathway of collagen.^22^, ^23^ Emerging studies demonstrate that SERPINH1 is closely associated with cancer development and may be referred to as a potential biomarker of tumours.^24^ Nevertheless, the function of SERPINH1 in ccRCC remains elusive.

In this study, we identified a circRNA derived from the transportin‐3 (TNPO3) gene (termed circ‐TNPO3). This was downregulated in ccRCC tissues and associated with ccRCC distant metastases, World Health Organization/International Society of Urologic Pathologists (WHO/ISUP) grade and tumour T stage. Functional assays indicated that circ‐TNPO3 suppressed the migration and proliferation of ccRCC cells both in vivo and in vitro. Mechanistically, circ‐TNPO3 could bind to IGF2BP2, thereby synergically downregulating SERPINH1 expression and inhibiting ccRCC migration. In conclusion, circ‐TNPO3 is a potential target for the treatment of patients with ccRCC.

## 2 MATERIALS AND METHODS

### 2.1 Human ccRCC specimens

A total of 114 pair ccRCC tissues and matched paracancerous tissues from ccRCC patients treated by radical nephrectomy or nephron‐sparing surgery were collected at the Southwest Hospital, Chongqing, China (2019–2021). Clinical characteristics are listed in Tables S1 and S2. All cases were confirmed by histopathological examination, and no patient received any neoadjuvant therapy. The surgically excised samples were collected in centrifuge tubes pre‐filled with RNA later and stored at −80°C. WHO/ISUP is a standard histological staging system for RCC based on the assessment of the nucleoli. T stage is the ‘T’ part of the TNM system to stage RCC and is the most important indicator for assessing the size and extension of the main tumour. Informed consent was received from each patient and the research design was granted by the Ethics Review Committee of Chongqing Medical University and Southwest Hospital, Army Medical University.

### 2.2 Microarray analysis

Four pairs of fresh ccRCC tissues and their matched paracancerous tissues were prepared. RNA extraction, quality identification and analysis of differentially expressed circRNAs were performed by Shanghai Genomics Corporation (Shanghai, China) as described previously.^25^, ^26^

### 2.3 RNA extraction and quantitative real‐time PCR (qRT‐PCR) analysis

TRIzol (Invitrogen, Carlsbad, CA) was applied to the extraction of RNA from ccRCC cell lines and tissues. Isolation and extraction of RNA from nucleus and cytoplasm were performed using a PARIS Kit (Invitrogen, Carlsbad, CA). RNA was reverse transcribed to cDNA using the PrimeScript RT Reagent Kit (Takara, Dalian, China). The expressions of circ‐TNPO3, IGF2BP2, SERPINH1 and other RNAs were analysed by quantitative real‐time (qRT)‐PCR with an SYBR Premix Ex Taq kit (Takara, Dalian, China). The expression of microRNA was measured using TaqMan MicroRNA Assays (Invitrogen, Carlsbad, CA). U6 or GAPDH was used as the normalizing control. The Bio‐Rad CFX96 real‐time PCR system and 2^−ΔΔ^ *^CT^* method were performed to evaluate the transcript levels. qPCR procedure was shown as follows: pre‐denaturation at 95°C for 30 s, denaturation at 95°C for 10 s, annealing at 58°C for 10 s and final extension at 72°C for 10 s. A total of 40 cycles were performed from denaturation to extension. The primer sequences information is given in Table S3.

### 2.4 RNA‐fluorescence in situ hybridization (FISH) assay

The Cy3‐labelled circ‐TNPO3 probes targeting the circ‐TNPO3 back splice site were synthesized at GenePharma (Shanghai, China). ccRCC cells were seeded on a culture plate (iBiDi, Martin Reid, Germany). Localization of circ‐TNPO3 in ccRCC cells was detected by RNA‐fluorescence in situ hybridization (FISH) assay using the FISH kit (GenePharma, Shanghai, China). Images were acquired with a Confocal Microscope (Leica Microsystems, Germany). The information on probes is presented in Table S4.

### 2.5 Cell transfection and lentiviral transduction

siRNA constructs targeting circ‐TNPO3, IGF2BP2, SERPINH1, epithelial splicing regulatory protein 1 (ESRP1) and their non‐targeting controls were synthesized by GenePharma (Shanghai, China). The sequence information on these siRNAs is listed in Table S5. In addition, the full‐length sequences of circ‐TNPO3, IGF2BP2 and SERPINH1 were cloned into the pCD‐ciR, pcDNA3.1 and pCDH vector, respectively, to obtain the corresponding pCD‐circ‐TNPO3, pcDNA3.1‐IGF2BP2 and pCDH‐SERPINH1 overexpression plasmids. Plasmids carrying the IGF2BP2 mutant and Flag sequences were constructed in the pcDNA3.1 vector background. Transfection of siRNAs or plasmids into ccRCC cells was conducted with Lipofectamine 2000 (Invitrogen, Carlsbad, CA) or Neofect DNA transfection reagent (Neofect, Beijing, China). To construct stable cell lines, we ligated the shRNA targeting circ‐TNPO3 into the HBLV‐LUC‐PURO vector and synthesized lentiviruses at HanBio Biotechnology Co., Ltd (Shanghai, China). The sequences of lentiviruses are shown in Table S5. Next, Caki‐1, RCC‐JF and 786‐O cells were infected with lentivirus constructs encoding circ‐TNPO3 shRNA at a multiplicity of infection 1:50 for 2 weeks with puromycin (2–3 μg/ml). The qRT‐PCR assay verified the efficiency of knockdown, and these cells were used for subsequent experiments.

### 2.6 RNA pull‐down assay

The interaction of circ‐TNPO3 with candidate proteins and microRNAs was verified by an RNA pull‐down assay. We constructed a plasmid containing full‐length circ‐TNPO3 and MS2bs sequences in the pLC5‐ciR vector background. The MS2 binding sites sequence is as follows: TACTCCTAGTGGGTACATGGAAGGGACTAGT GGGTACAGACGTCCAGCTGAGATCTTTTGTACTCCTAGTGGGTACATG. The pLC5‐circ‐TNPO3‐MS2 and pMS2‐GFP (Addgene, MA, USA) plasmids were then co‐transfected into HEK293T cells. The RNA immunoprecipitation (RIP) assay was conducted with GFP antibody, which could specifically pull‐down endogenous RNAs and proteins associated with circ‐TNPO3; The negative control is IgG.

### 2.7 RIP assay

RIP experiments were performed with the Magna RIP Kit (Millipore, MA, USA). The cells were processed at 1 × 10^7^ cells/reaction density and lysed by adding lysis buffer containing protease and RNase inhibitors for 5 min. The magnetic beads were combined with 5‐μg antibody against IGF2BP2 (Millipore, MA, USA), N6‐Methyladenosine (CST, Boston, USA) or Flag (CST, Boston, USA), or control IgG at indoor temperature, and the corresponding cell lysates were added; the samples were incubated overnight at 4°C. The samples were subjected subsequently to RNA purification and protein extraction, and the RIP efficiency was verified by Western blot assay; the enrichment of circRNAs or mRNAs was verified by qRT‐PCR assay.

### 2.8 Immunofluorescence and immunohistochemistry assays

To perform an immunofluorescence assay, ccRCC cells were grown on a μ‐Slide 8 Well (iBiDi, Martin Reid, Germany) culture plate and incubated with antibodies specific to IGF2BP2 (Millipore, MA, USA) overnight at 4°C. Further, the cells were stained using DAPI (300 nmol/L) and IgG antibody (Alexa Fluor 488, 1:400). For immunohistochemistry assay, paraffin sections were dewaxed and hydrated, following which they were incubated with the corresponding antibody overnight at 4°C. Subsequently, the sections were stained using haematoxylin and DAB. The antibodies used for immunohistochemical experiments were as follows: Ki67 (1:500, Servicebio, Wuhan, China), SERPINH1 (1:100, SAB, Maryland, USA), IGF2BP2 (1:200, Abcam, Cambridge, UK), SLUG (1:100, SAB, Maryland, USA) and SNAIL (1:200, Bioss, Beijing, China).

### 2.9 RNA sequencing

To detect differentially expressed genes in Caki‐1 cells after IGF2BP2‐RIP and circ‐TNPO3 silencing, RNA sequencing was performed by Shanghai Genomics Corporation (Shanghai, China), including library construction and computational analysis. To screen the target genes of IGF2BP2, total RNAs of Caki‐1 cells with IGF2BP2 knockdown or controls were isolated. mRNA sequencing was carried out by Tsingke Biotechnology Co., Ltd (Beijing, China).

### 2.10 Statistical analysis

Statistical analysis was performed via GraphPad Prism 8 software. Student's *t*‐test, Fisher's exact test, chi‐square test or Mann–Whitney *U* test were conducted to verify the differences among the two groups. Pearson correlation analysis was applied to determine the correlation between the two groups. Differences were considered statistically significant when *p *< .05.

## 3 RESULTS

### 3.1 Circ‐TNPO3 is downregulated in ccRCC and its low level significantly correlates with distant metastasis, WHO/ISUP grade and tumour T stage

**FIGURE 1:**
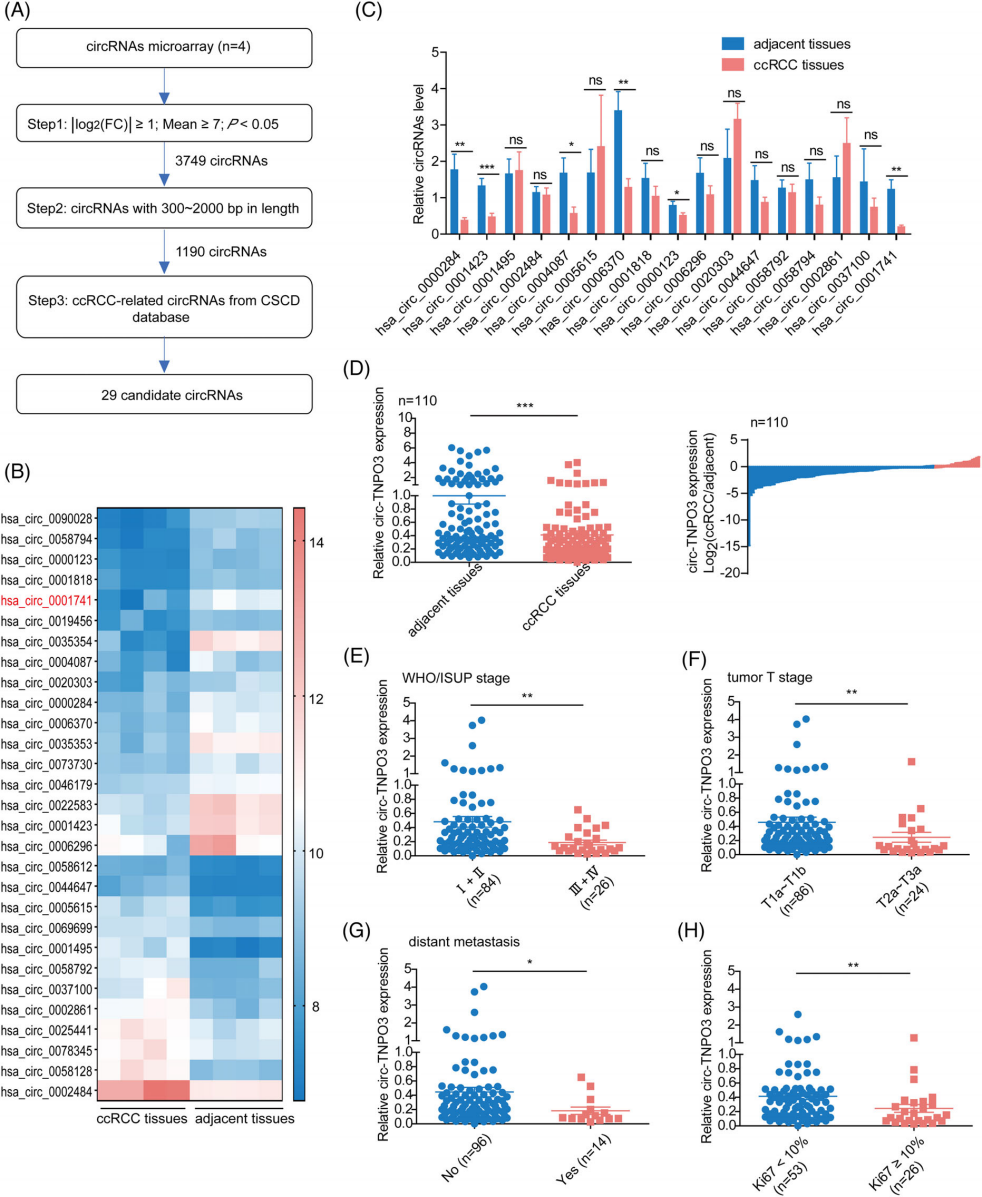
Circ‐transportin‐3 (TNPO3) is downregulated in clear cell renal cell carcinoma (ccRCC) and its low level significantly correlates with distant metastasis, World Health Organization/International Society of Urologic Pathologists (WHO/ISUP) grade and tumour T stage. (A) Flow chart illustrating the screening criteria of circular RNAs (circRNAs) in ccRCC. (B) A cluster heat map showing 29 upregulated and downregulated circRNAs. Vertical coordinates indicate circRNAs, whereas horizontal coordinates indicate samples. Red represents upregulated, whereas blue indicates downregulated circRNAs. (C) Quantitative real‐time (qRT)‐PCR assays for the expression of candidate circRNAs in ccRCC tissues. Paired *t*‐test, *n* = 15. ns, not significant. * *p *< .05, **p < .01, ***p < .001. (D) Left: qRT‐PCR analysis of circ‐TNPO3 expression in 110 pairs of ccRCC tissues and matched adjacent noncancerous tissues. Right: A decrease in circ‐TNPO3 expression is observed in 80% of the ccRCC patients. Paired *t*‐test, *n* = 110. ***p < .001. (E–G) circ‐TNPO3 expression in ccRCC patients with different WHO/ISUP stages. (E) (Ⅰ and Ⅱ [*n* = 84] vs. III and IV [*n* = 26)]), tumour size (F) (T1a–T1b [*n* = 86] vs. T2–T4 [*n* = 24]) and distant metastases (G) (No [*n* = 96] vs. Yes [*n* = 14]). (H) Correlation between circ‐TNPO3 expression and Ki67 level in ccRCC (Ki67 < 10% [*n* = 53] vs. Ki67 ≥ 10% [*n* = 26]). Mann–Whitney *U* test, *p < .05, **p < .01

To determine the expression of circRNAs in ccRCC tissues, circRNA microarray analysis was performed in four matched ccRCC tissues and adjacent noncancerous tissues. By using │log_2_FC│≥1, average fluorescence signal value ≥7, and *p* < .05 as the cut‐off criteria, 3749 differentially expressed circRNAs were identified, including 2210 upregulated and 1539 downregulated circRNAs between ccRCC and matched noncancerous tissues (Figure S1). We included circRNAs of 300–2000‐bp length for further analysis and identified a total of 1190 differentially expressed circRNAs (Table S6). In comparing the previously mentioned candidate circRNAs with the list of disease‐related circRNAs from the cancer‐specific circRNAs database (http://gb.whu.edu.cn/CSCD/), 12 upregulated and 17 downregulated circRNAs were identified (Figure 1A,B and Table S7). Excluding some circRNAs that are difficult to design PCR primers, we finally selected 17 candidate circRNAs for subsequent validation. The expression of these circRNAs in 15 pairs of ccRCC tissues was verified by qRT‐PCR analysis. A total of six downregulated circRNAs, including has_circ_0000284, hsa_circ_0001423, has_circ_0004087, has_circ_0006370, has_circ_0000123 and hsa_circ_0001741, were identified, among which circ‐TNPO3 (hsa_circ_0001741) (fold change = .16, *p* < .01) was the most dysregulated according to its fold change value (Figure 1C).

Next, we analysed the level of circ‐TNPO3 in a larger cohort comprising ccRCC tissues. The downregulated expression of circ‐TNPO3 was validated between 110 paired ccRCC tissues and matched noncancerous tissues (fold change = .41, *p *< .001). The decrease in circ‐TNPO3 level was observed in 89 (80%) of the 110 ccRCC tissues relative to the correspondingly matched noncancerous tissues (Figure 1D). However, there was no remarkable difference in the level of parent TNPO3 in ccRCC tissues (Figure S2). We further investigated whether the expression of circ‐TNPO3 was related to the clinical characteristics of ccRCC. We grouped the ccRCC sample cohort (*n* = 110) according to the WHO/ISUP stage, tumour T stage and distant metastasis to compare the circ‐TNPO3 levels among these groups. The results suggested that lower levels of circ‐TNPO3 were associated with ccRCC of higher WHO/ISUP grade (I–II [*n* = 84] vs. III–IV [*n* = 26], *p *< .01), more advanced tumour T stage (T1a–T1b [*n* = 86] vs. T2a–T4 [*n* = 24], *p *< .01) and distant metastasis (Yes) (metastasis [No] [*n* = 96] vs. metastasis [Yes] [*n* = 14], *p *< .05) (Figure 1E–G). Moreover, the correlation between the level of circ‐TNPO3 expression and clinical immunohistochemical indexes indicated Ki67 expression in 79 of the 110 ccRCC patients. Subsequently, these 79 patients were divided into Ki67 low expression (Ki67 < 10%, *n* = 53) and high expression groups (Ki67 ≥ 10%, *n* = 26). Circ‐TNPO3 levels were significantly lower in the Ki67 ≥ 10% group relative to the Ki67 < 10% group (Figure 1H). The above results indicated that circ‐TNPO3 was downregulated in ccRCC and may act as a ccRCC suppressor.

### 3.2 Identification and characteristics of circ‐TNPO3 in ccRCC

**FIGURE 2:**
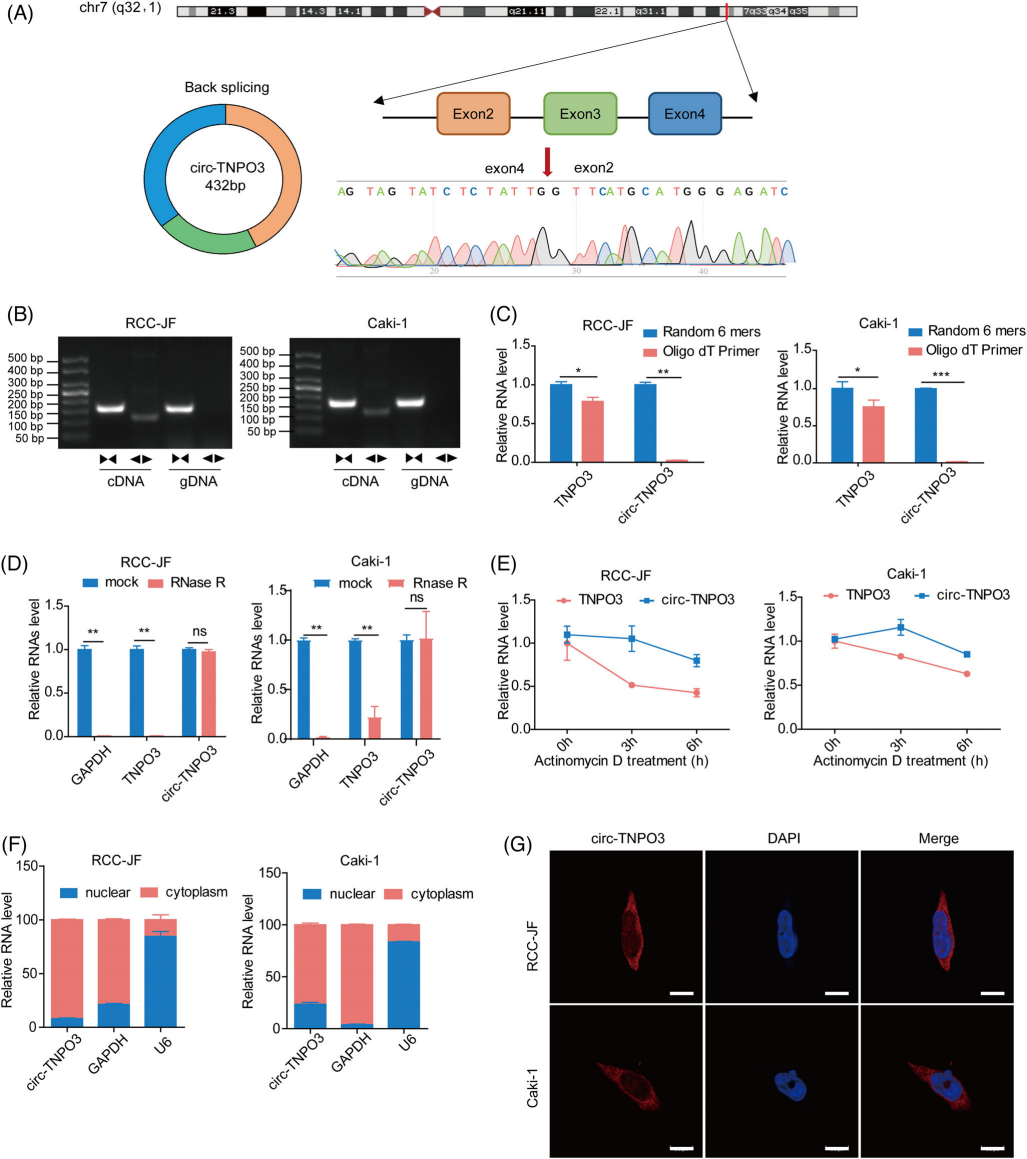
Identification and characteristics of circ‐transportin‐3 (TNPO3) in clear cell renal cell carcinoma (ccRCC). (A) Schematic illustration showing circ‐TNPO3 contains three exons (432 bp) from host gene TNPO3. The specific primers of circ‐TNPO3 were validated by quantitative real‐time (qRT)‐PCR and its sequence was proven by DNA sequencing. (B) The circulation of circ‐TNPO3 from cDNA and gDNA was verified via qRT‐PCR using divergent and convergent primers in Caki‐1 and RCC‐JF cell lines. (C) qRT‐PCR analysis of circ‐TNPO3 and linear TNPO3 in total RNA reverse transcribed by random 6 mers or oligo dT 18 primers. (D) Effect of RNase R treatment on the expression of circ‐TNPO3 and linear TNPO3 in Caki‐1 and RCC‐JF cells were determined via qRT‐PCR. (E) qRT‐PCR assay to detect the stabilities of circ‐TNPO3 and linear TNPO3 after actinomycin D treatment in Caki‐1 or RCC‐JF cells. (F and G) The nucleocytoplasmic fractionation and fluorescence in situ hybridization (FISH) assays were used to detect the subcellular localization of circ‐TNPO3. Red dots represent positive signals, and the nuclei were stained with DAPI. Scale bar, 10 μm. The data were all presented as the mean ± SD. Student's *t*‐test, *n* = 3. *p < .05, **p < .01, ***p < .001

circ‐TNPO3 (chr7: 128655032–128658211), located on chromosome 7, is derived from exon 2 to exon 4 (432 bp) of the host gene TNPO3 via back‐splicing. By designing divergent primers and Sanger sequencing, we confirmed the predicted junction of circ‐TNPO3 (Figure 2A). To verify the characterization of circ‐TNPO3, we first designed specific primers to amplify linear and circular TNPO3 sequences. qRT‐PCR analysis suggested that circ‐TNPO3 could only be amplified from the cDNA template using divergent primers but not gDNA, whereas linear TNPO3 mRNA could be detected from both cDNA and gDNA templates using convergent primers (Figure 2B). Next, we only amplified circ‐TNPO3 using random primers but not oligo (dT)18 primers from RCC‐JF and Caki‐1 cells (Figure 2C). Circ‐TNPO3 was resistant to RNase R treatment relative to linear TNPO3 and GAPDH (Figure 2D). Subsequently, qRT‐PCR analysis after actinomycin D treatment in RCC‐JF and Caki‐1 cells indicated that circ‐TNPO3 had higher stability than linear TNPO3 (Figure 2E). In addition, nucleoplasmic separation and FISH assays showed that circ‐TNPO3 was predominantly localized to the cytoplasm (Figure 2F,G). Taken together, the above results showed that circ‐TNPO3 was a stable circRNA in ccRCC.

### 3.3 Circ‐TNPO3 suppresses migration and proliferation properties of ccRCC cells in vitro

**FIGURE 3:**
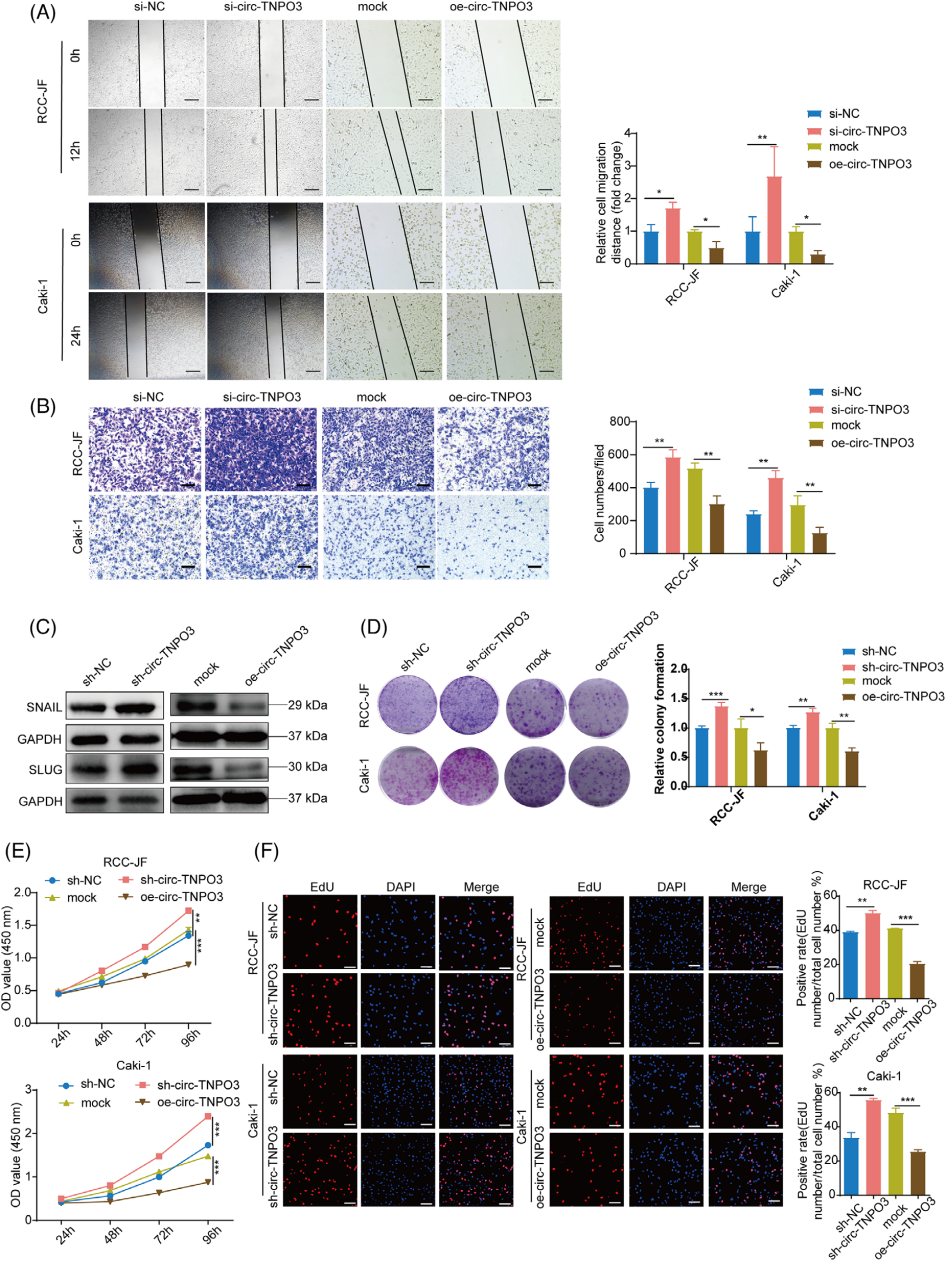
Circ‐transportin‐3 (TNPO3) inhibits the migration and proliferation of clear cell renal cell carcinoma (ccRCC) cells in vitro. (A) Migration ability was identified by wound healing assay after knockdown or overexpression of circ‐TNPO3 in Caki‐1 and RCC‐JF cells. Scale bar, 50 μm. The data were showed as the mean ± SD. Student's *t*‐test, *n* = 3. *p < .05, **p < .01. (B) Transwell assays were performed after knockdown or overexpression of circ‐TNPO3 in Caki‐1 and RCC‐JF cells. Scale bar, 50 μm. Student's *t*‐test, *n* = 3. *p < .05, ***p < .001. (C) The protein levels of SNAIL and SLUG in Caki‐1 cells with overexpression or stable knockdown of the circ‐TNPO3. (D–F) Proliferation ability was examined by colony formation, CCK‐8 and EdU assays after stable silence or overexpression of circ‐TNPO3 in the Caki‐1 and RCC‐JF cells. Scale bar, 50 μm. Data were presented as mean ± SD. Student's *t*‐test, *n* = 3. *p < .05, **p < .01, ***p < .001

In order to identify the potential function of circ‐TNPO3, we first constructed siRNAs which can target the back‐splice junction and overexpression vector of circ‐TNPO3. Then we confirmed successful interference and overexpression of circ‐TNPO3 in RCC‐JF and Caki‐1 cells; the mRNA and protein levels of linear TNPO3 remained unaffected (Figure S3A,B). The results illustrated that knocking down promoted but the overexpression of circ‐TNPO3 inhibited the migration of RCC‐JF and Caki‐1 cells (Figure 3A,B). We further examined whether circ‐TNPO3 inhibited ccRCC cell migration by regulating the epithelial–mesenchymal transition (EMT) pathway. We detected the influence of circ‐TNPO3 on the expression of five EMT‐related proteins (E‐cadherin, N‐cadherin, vimentin, SNAIL and SLUG). The results showed that inhibition significantly increased, whereas the overexpression of circ‐TNPO3 reduced the levels of SNAIL and SLUG (Figure 3C). However, circ‐TNPO3 had no significant influence on the expression of other EMT‐related proteins (Figure S4).

To investigate the impact of circ‐TNPO3 on cellular proliferation, we established lentivirus‐based stable sh‐circ‐TNPO3‐Caki‐1 and sh‐circ‐TNPO3‐RCC‐JF cell lines (Figure S3C,D). Colony formation, CCK‐8 and EdU assays confirmed that stable inhibition significantly boosted, whereas the overexpression of circ‐TNPO3 reduced the proliferation of ccRCC cells (Figure 3D–F). Therefore, our data suggested that circ‐TNPO3 inhibited the migration and proliferation of ccRCC cells in vitro.

### 3.4 Circ‐TNPO3 inhibits tumourigenesis and metastasis of xenograft tumours in vivo

**FIGURE 4:**
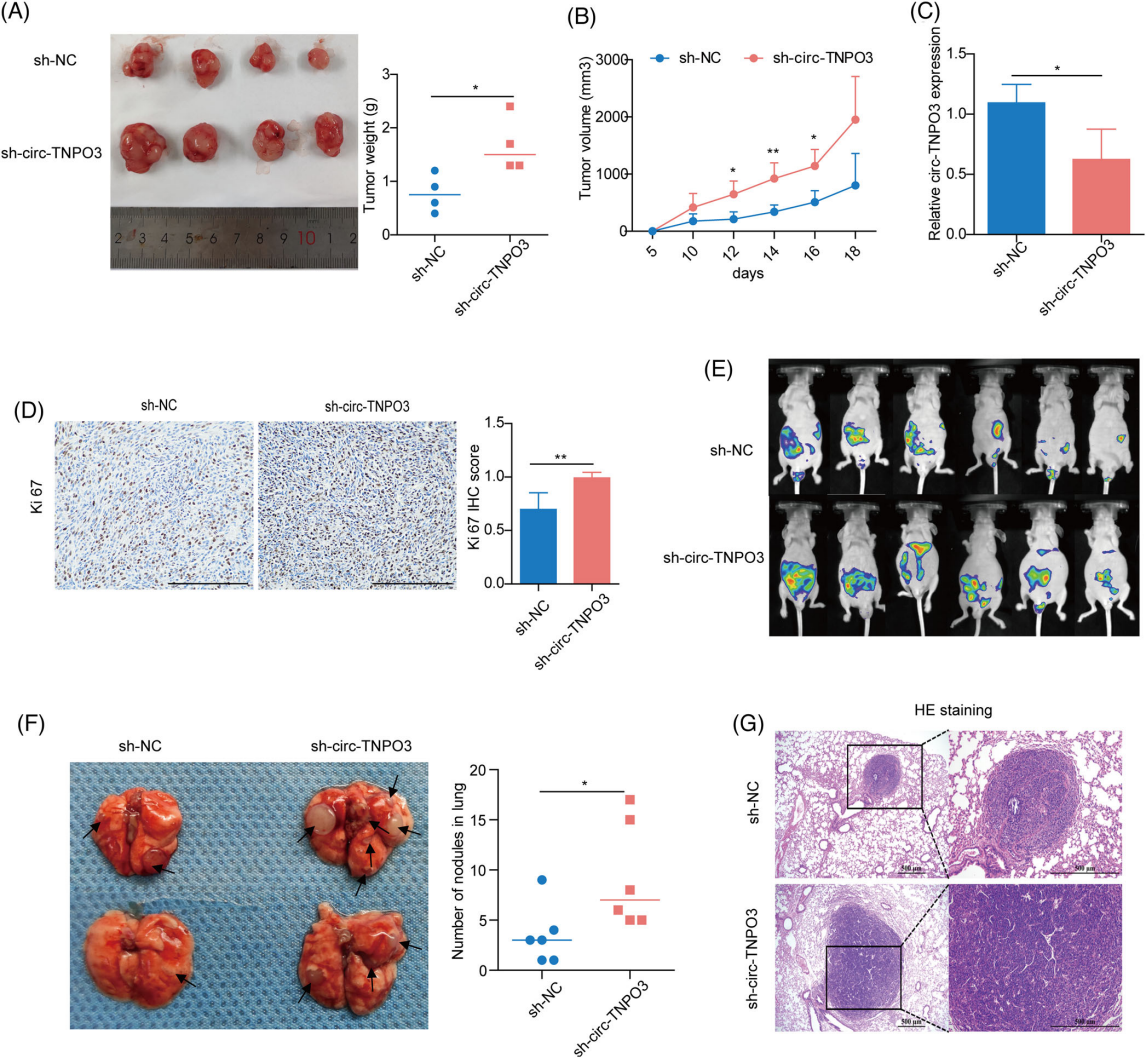
Circ‐transportin‐3 (TNPO3) inhibits tumourigenesis and metastasis of xenograft tumours in vivo. (A) Left: The subcutaneous xenografts of sh‐NC and sh‐circ‐TNPO3 cells in implanted in nude mice. Right: The weight of xenografts at indicated time. Student's *t*‐test, *n* = 4. *p < .05. (B) The changes in tumour volume were recorded every 2 days in nude mice. Student's *t*‐test, *n* = 4. *p < .05, **p < .01. (C) The level of circ‐TNPO3 in tumour tissues of nude mice was measured by quantitative real‐time (qRT)‐PCR. Data were showed as mean ± SD. Student's *t*‐test, *n* = 4. *p < .05. (D) The protein expression of Ki67 was evaluated by IHC assay; scale bar, 250 μm. Mann–Whitney *U* test, *n* = 6. **p < .01. (E) Representative images of the abdominal dissemination model. (F) Representative images of mice lungs in a nude mouse lung metastasis model (left) and the quantification of lung metastatic colonization (right). Mann–Whitney *U* test, *n* = 6. *p < .05. (G) Representative images of pulmonary metastatic nodules after H&E staining in lung metastasis model. Scale bar, 500 μm

To further elucidate the function of circ‐TNPO3 in an animal model of a tumour, we generated a 786‐O cell line with stably knocked‐down circ‐TNPO3 as previously described. We confirmed the knockdown of circ‐TNPO3 in sh‐circ‐TNPO3‐786‐O cells, and these cells showed increased migration ability (Figure S3E,F). In the nude mice xenograft model, the inhibition of circ‐TNPO3 evidently enhanced the growth rate and volume of subcutaneous tumour xenografts (Figure 4A,B). We validated the knockdown efficiency of circ‐TNPO3 in the sh‐circ‐TNPO3 group of xenografted mice (Figure 4C). Moreover, IHC assays demonstrated that the decreased expression of circ‐TNPO3 was associated with a higher expression of Ki67 in xenografts (Figure 4D). In the abdominal dissemination model, the ability of intra‐abdominal tumour dissemination was significantly enhanced in nude mice injected with sh‐circ‐TNPO3‐Caki‐1 cells as compared to the controls (Figure 4E). In the pulmonary metastasis model of ccRCC, we found that nude mice injected with sh‐circ‐TNPO3‐786‐O cells showed an increased number of metastatic pulmonary nodules as compared to the control group (Figure 4F,G). These results suggested that circ‐TNPO3 could suppress the growth and metastasis of ccRCC in vivo.

### 3.5 Circ‐TNPO3 can directly interact with the RNA binding protein, IGF2BP2, in ccRCC cells

**FIGURE 5:**
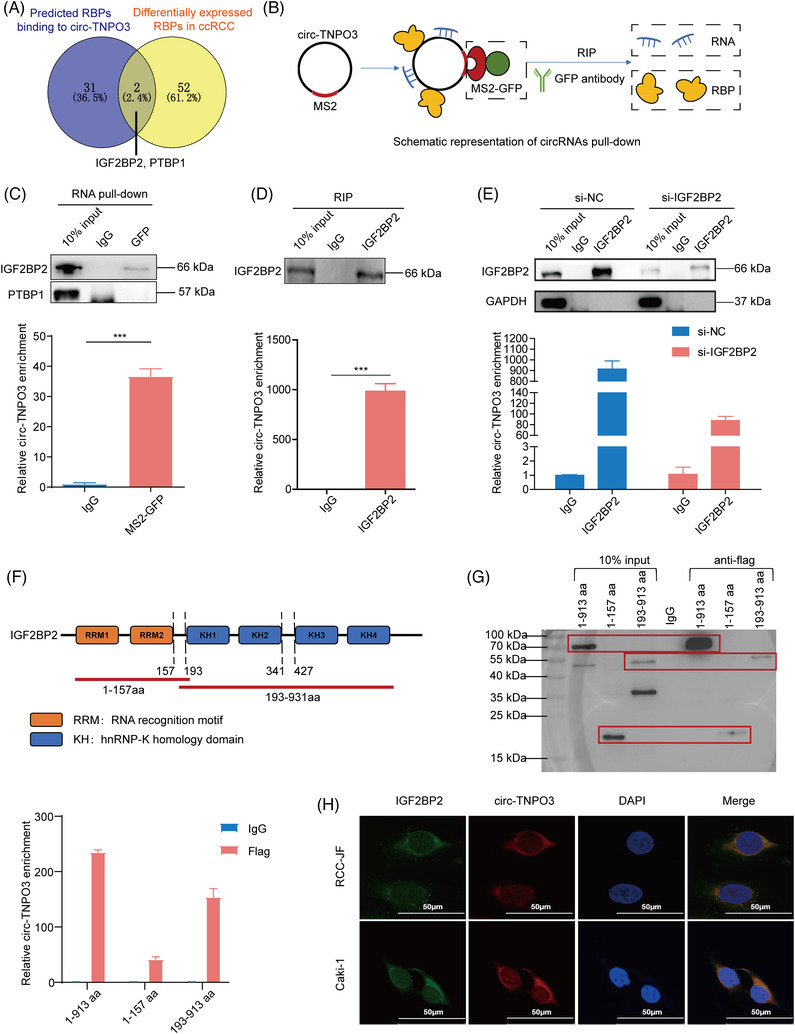
Circ‐transportin‐3 (TNPO3) directly interacted with insulin‐like growth factor 2 mRNA‐binding protein 2 (IGF2BP2) protein in clear cell renal cell carcinoma (ccRCC) cells. (A) Overlap of circ‐TNPO3‐binding RBPs predicted by Starbase website and the expression analysis in ccRCC from TCGA database and literature. (B) Schematic diagram of circ‐TNPO3 pulls down in 293T cells. (C) The efficiency of circ‐TNPO3 pulled down by RNA immunoprecipitation (RIP) assay was examined through quantitative real‐time (qRT)‐PCR. Data were presented as mean ± SD. Student's *t*‐test, *n* = 2. ***p < .001. Western blot experiments examined the binding ability of circ‐TNPO3 to IGF2BP2 or PTBP1 in 293T cells. (D and E) Western blot assays identified the efficiency of RIP assay with or without a knockdown of the IGF2BP2. The level of circ‐TNPO3 enriched by IGF2BP2 was detected by qRT‐PCR. Data were presented as mean ± SD. Student's *t*‐test, *n* = 2. ***p < .001. (F) Structural diagram of IGF2BP2 protein and truncated IGF2BP2 variants. (G) The full‐length or truncated mutant of IGF2BP2 variants was pulled down by anti‐flag which was verified by Western blot. The expression of enriched circ‐TNPO3 in various groups was tested by qRT‐PCR. (H) The localization of circ‐TNPO3 (red) and IGF2BP2 (green) was detected by immunofluorescence and RNA‐fluorescence in situ hybridization (FISH) in RCC‐JF and Caki‐1 cells. Scale bar, 50 μm

Emerging evidence demonstrates that circRNAs play key roles as miRNA sponges, RNA‐binding protein scaffolds and translation. We first tested whether circ‐TNPO3 could function as an miRNA sponge. Several miRNA‐binding sites of circ‐TNPO3 were predicted using the Starbase and circRNA interactome (https://circinteractome.nia.nih.gov/) databases. Next, six candidate miRNAs were identified through the overlap of predicted results and expression analysis of miRNAs in ccRCC using TCGA database. However, we could not confirm the binding between candidate miRNAs and circ‐TNPO3 by luciferase reporter assays and RNA pull‐down assays (Figure S5A‐C). Next, analysis using the circRNADb database suggested that circ‐TNPO3 had a very low probability of encoding proteins. Finally, to examine whether circ‐TNPO3 could bind to proteins in ccRCC, we predicted 33 candidate RBPs using the Starbase website (CLIP data ≥ 1) (Table S8). Among the candidate RBPs, IGF2BP2 and PTBP1 were the only two that showed a differential expression in ccRCC tissues based on TCGA analysis and literature review (Figure 5A) (Table S9). Therefore, we chose IGF2BP2 and PTBP1 for further validation. Subsequently, the RNA pull‐down assay showed that IGF2BP2, but not PTBP1, could be pulled down by circ‐TNPO3 in 293T cells (Figure 5B,C). RIP assay and qRT‐PCR analysis further validated that IGF2BP2 could bind specifically to endogenous circ‐TNPO3 in Caki‐1 cells (Figure 5D). Moreover, the levels of circ‐TNPO3 which interacted with IGF2BP2 decreased significantly in ccRCC cells transfected with siRNA for IGF2BP2 (Figure 5E). Given that IGF2BP2 contains four hnRNP K homology (KH) domains and two RNA recognition motifs (RRMs), we constructed full‐length and truncated IGF2BP2 expression plasmids, to locate the binding sites of IGF2BP2 targeted by circ‐TNPO3. The RIP experiments indicated that circ‐TNPO3 bound with both the RRM (1–157 aa) and KH regions (193–913 aa) of IGF2BP2 but predominantly bound with the KH domain (193–913 aa) (Figure 5F,G). In condition, we found that the combined circ‐TNPO3 level was reduced if we divided the complete KH domain into two segments, KH 1 + 2 (193–341 aa) or KH 3 + 4 (427–913 aa), indicating that the integrity of the four KH domains may be critical for binding with circ‐TNPO3 (Figure S6A,B). Consistently, the enrichment of circ‐TNPO3‐IGF2BP2 complexes in the cytoplasm of ccRCC cells was observed by immunofluorescence and FISH assays (Figure 5H). Although circ‐TNPO3 was able to bind to IGF2BP2, we verified that circ‐TNPO3 and IGF2BP2 had no effect on each other's expression (Figure S7A,B). Moreover, the nucleoplasmic separation assay demonstrated that circ‐TNPO3 did not influence the subcellular localization of IGF2BP2 (Figure S7C). Overall, the above data indicated that circ‐TNPO3 could directly interact with the IGF2BP2 protein.

### 3.6 IGF2BP2 is downregulated in ccRCC and IGF2BP2–circ‐TNPO3 interaction is involved in inhibiting tumour migration induced by circ‐TNPO3 or IGF2BP2

**FIGURE 6:**
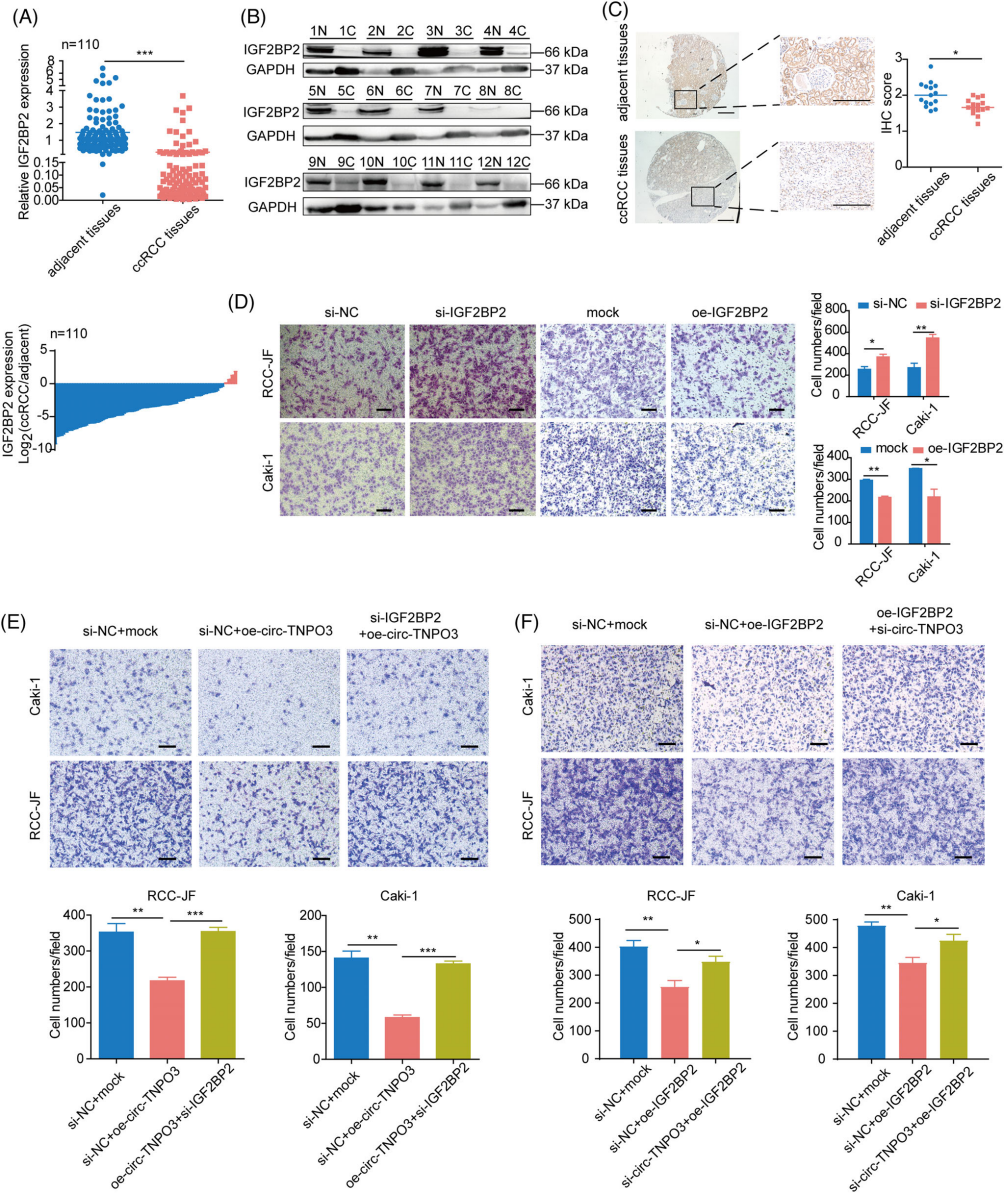
Insulin‐like growth factor 2 mRNA‐binding protein 2 (IGF2BP2) is downregulated in clear cell renal cell carcinoma (ccRCC) and is involved in inhibiting tumour migration induced by circ‐transportin‐3 (TNPO3). (A) Quantitative real‐time (qRT)‐PCR verified the expression of IGF2BP2 in ccRCC tissues (*n* = 110). Paired *t*‐test, *n* = 110. ***p < .001. (B and C) The protein levels of IGF2BP2 in ccRCC tissues were measured via Western blot and immunohistochemistry assays. Scale bar, 500 μm (left), 250 μm (right). Mann–Whitney *U* test, *n* = 15. *p < .05. (D) Transwell assay manifested the migration of RCC‐JF and Caki‐1 cells with overexpression or knockdown of IGF2BP2. Scale bar, 50 μm. Student's *t*‐test, *n* = 3. * *p *< .05, **p < .01. (E and F) Representative images (up) and quantification (down) in rescue assay showing the migration of Caki‐1 and RCC‐JF cells upon overexpression or knockdown of circ‐TNPO3 combined with IGF2BP2 knockdown or overexpression. Scale bar, 50 μm. Student's *t*‐test, *n* = 3. *p < .05, **p < .01, ***p < .001

IGF2BP2 is an RNA‐binding protein involved in tumour progression. However, its role in ccRCC remains unclear. We first detected the levels of IGF2BP2 in ccRCC tissues compared to those in matched adjacent noncancerous tissues (*n* = 110) and found that it was significantly downregulated in human ccRCC tissues (Figure 6A). The decrease in IGF2BP2 expression was observed in 103 (93%) of the 110 ccRCC tissues. Furthermore, Western blotting and IHC assay also verified the downregulation of IGF2BP2 protein in ccRCC tumour tissues compared with matched adjacent noncancerous tissues (Figure 6B,C). To further elucidate the potential anti‐oncogenic role of IGF2BP2 in ccRCC, we transfected ccRCC cell lines with siRNA or overexpression plasmids of IGF2BP2 (Figure S8A,B). Transwell assay suggested that silencing promoted, whereas the overexpression of IGF2BP2 inhibited the migration of ccRCC cells (Figure 6D). More importantly, we confirmed that the interference of IGF2BP2 expression significantly rescued the circ‐TNPO3‐mediated inhibition of ccRCC cell migration ability (Figure 6E). In addition, silencing circ‐TNPO3 could rescue the decreased migratory potential induced upon the overexpression of IGF2BP2. Circ‐TNPO3 could also mediate the inhibition of ccRCC cell migration induced by IGF2BP2 (Figure 6F). Therefore, the above findings suggested that IGF2BP2 could suppress the migration of ccRCC cells in vitro, and circ‐TNPO3 may be involved in the functioning of IGF2BP2. Synergistically, IGF2BP2 may be involved in inhibiting tumour migration induced by circ‐TNPO3.

### 3.7 Circ‐TNPO3 downregulates SERPINH1 expression by interacting with IGF2BP2 and decreasing the stability of SERPINH1 mRNA

**FIGURE 7:**
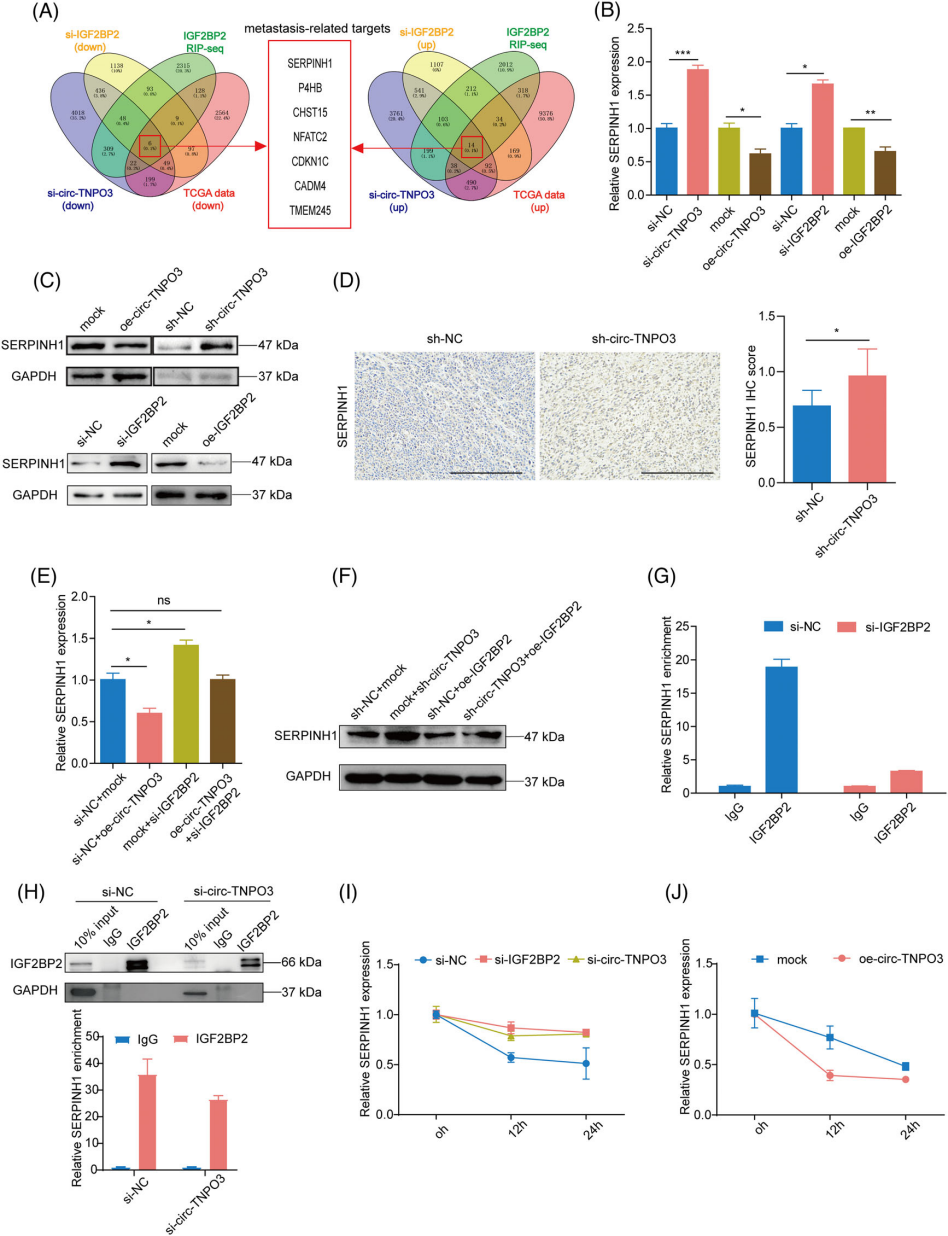
Circ‐transportin‐3 (TNPO3) downregulates serpin family H member 1 (SERPINH1) expression via interacting with insulin‐like growth factor 2 mRNA‐binding protein 2 (IGF2BP2) and decreasing the stability of SERPINH1 mRNA. (A) Venn diagram showing the strategy to screen potential targets of circ‐TNPO3‐IGF2BP2 axis. (B and C) The mRNA (B) and protein (C) levels of SERPINH1 were measured in Caki‐1 cells overexpressing or inhibiting of circ‐TNPO3 or IGF2BP2. Data were shown as mean ± SD. Student's *t*‐test, *n* = 3. *p < .05, **p < .01, ***p < .001. (D) The protein level of SERPINH1 in the xenograft mouse model was tested by IHC assay; scale bar, 250 μm. Mann–Whitney *U* test, *n* = 6. *p < .05. (E) The mRNA levels of SERPINH1 in Caki‐1 cells transfected with OE‐circ‐TNPO3, si‐IGF2BP2 or OE‐circ‐TNPO3+si‐IGF2BP2 were tested by quantitative real‐time (qRT)‐PCR. Data were shown as mean ± SD. Student's *t*‐test, *n* = 3. ns, not significant, *p < .05. (F) The protein levels of SERPINH1 in Caki‐1 cells stably transfected with sh‐NC or sh‐circ‐TNPO3, and co‐transfected with OE‐IGF2BP2 or control were measured by Western blot assay. (G and H) The enrichment of SERPINH1 pulled down by IGF2BP2 protein in Caki‐1 cells with or without knockdown the IGF2BP2 (G) or circ‐TNPO3 (H) was analysed by qRT‐PCR. (I and J) The actinomycin D assay shows the stability of SERPINH1 in Caki‐1 cells with IGF2BP2 knockdown (I) and circ‐TNPO3 knockdown or overexpression (I and J) at the indicated time point.

To further investigate the exact mechanism through which circ‐TNPO3‐IGF2BP2 regulated ccRCC migration, we applied RNA‐seq of Caki‐1 cells transfected with si‐circ‐TNPO3 and si‐IGF2BP2 (Tables S10 and S11). On overlapping the above RNA‐Seq data with IGF2BP2 RIP‐Seq data (Table S12) and gene expression analysis for ccRCC from TCGA database, 20 candidate genes were identified. Based on the functions of the above genes, seven metastasis‐related genes were selected, including SERPINH1, P4HB, CHST15, NFATC2, CDKN1C, CADM4 and TMEM245, as candidate targets of the circ‐TNPO3‐IGF2BP2 axis (Figure 7A). Next, we evaluated the expressions of the above target genes in Caki‐1 cells by inhibiting or overexpressing circ‐TNPO3/IGF2BP2. Interestingly, the inhibition of circ‐TNPO3 or IGF2BP2 dramatically increased, yet their overexpression reduced the expression of SERPINH1 (Figure 7B,C). However, circ‐TNPO3 or IGF2BP2 showed no consistent influence on the expression of other candidate mRNAs (Figure S9A,B). In addition, IHC results showed that inhibition of circ‐TNPO3 also increased the level of SERPINH1 in the xenograft mouse model (Figure 7D). Next, rescue experiment results demonstrated that the knockdown of IGF2BP2 attenuated OE‐circ‐TNPO3‐induced SERPINH1 downregulation (Figure 7E). As expected, the overexpression of IGF2BP2 significantly counteracted si‐circ‐TNPO3‐mediated increased expression of SERPINH1 (Figure 7F). Considering that IGF2BP2 is an important RNA‐binding protein which can bind with the target mRNA and affect its stability, we hypothesized that the circ‐TNPO3–IGF2BP2 complex may regulate the expression of SERPINH1 by influencing its mRNA stability. CLIP‐seq data predicted IGF2BP2‐SERPINH1 interaction.^27^ We validated the interaction between IGF2BP2 protein and SERPINH1 mRNA in RIP assays. Moreover, the knockdown of IGF2BP2 or circ‐TNPO3 correspondingly attenuated the interaction between IGF2BP2 and SERPINH1 (Figure 7G,H). Next, an mRNA decay assay for SERPINH1 was performed. The inhibition of IGF2BP2 or circ‐TNPO3 significantly improved the stability of SERPINH1 mRNA in Caki‐1 cells (Figure 7I). Conversely, SERPINH1 mRNA decay rates were accelerated after circ‐TNPO3 overexpression (Figure 7J). Collectively, these results suggested that circ‐TNPO3 downregulated SERPINH1 expression by interacting with IGF2BP2, thereby decreasing the stability of SERPINH1 mRNA.

### 3.8 Circ‐TNPO3 suppresses migration of ccRCC cells in a SERPINH1‐dependent manner

**FIGURE 8:**
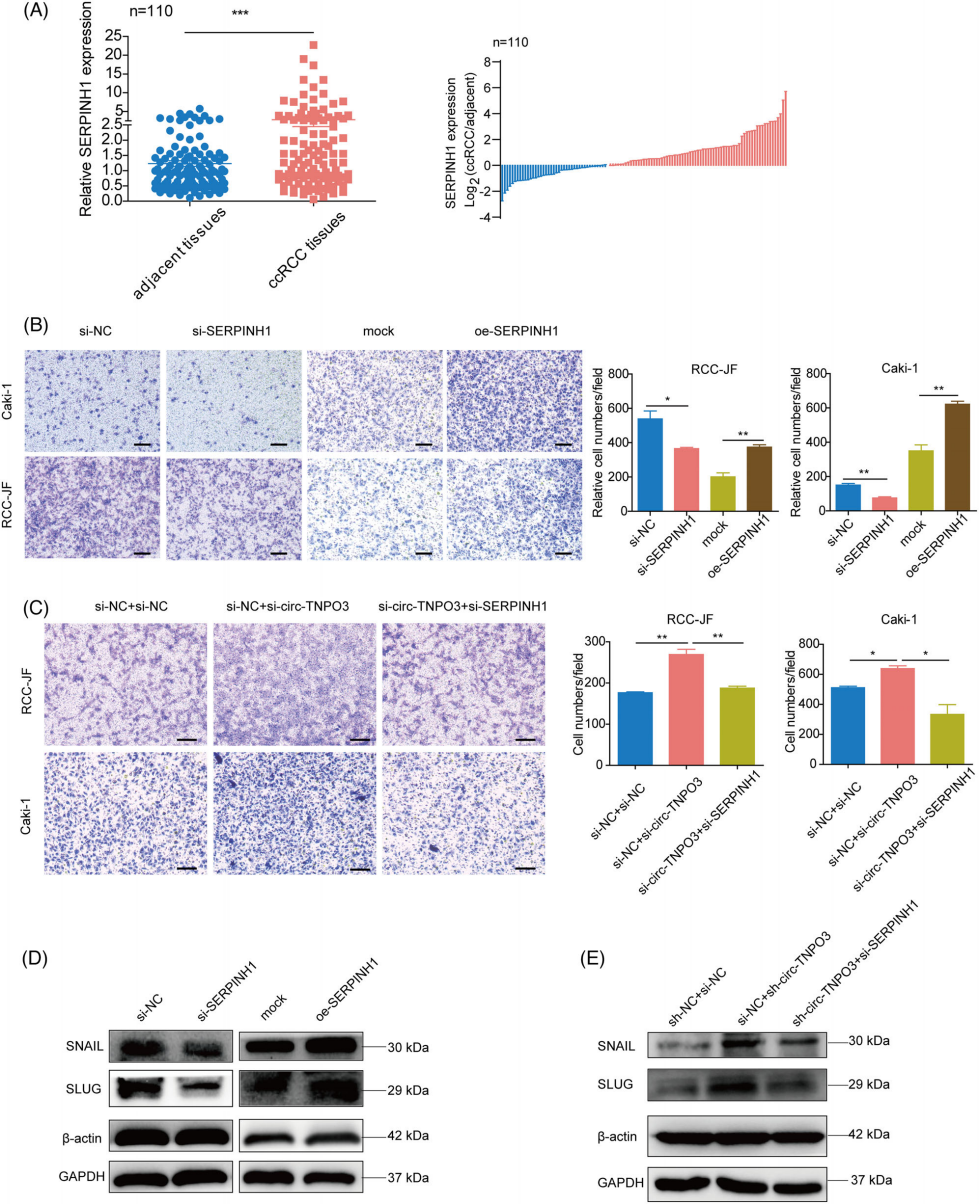
Circ‐transportin‐3 (TNPO3) suppresses the migration of clear cell renal cell carcinoma (ccRCC) in a serpin family H member 1 (SERPINH1)‐dependent manner. (A) Quantitative real‐time (qRT)‐PCR analysed the expression of SERPINH1 in ccRCC tissues. Paired *t*‐test, *n* = 110. ***p < .001. (B) Transwell assay indicated the migration of RCC‐JF and Caki‐1 cells with the overexpression or knockdown of SERPINH1. Scale bar, 50 μm. Student's *t*‐test, *n* = 3. *p < .05, **p < .01. (C) Transwell assay showed the migration of Caki‐1 and RCC‐JF cells upon knockdown of circ‐TNPO3 combined with SERPINH1 knockdown. Scale bar, 50 μm. Student's *t*‐test, *n* = 3. *p < .05, **p < .01. (D) Western blot analysis showed the protein levels of SNAIL and SLUG in Caki‐1 cells with knockdown or overexpression of the SERPINH1. (E) Western blot assay shows the protein levels of SNAIL and SLUG in Caki‐1 cells stably transfected with sh‐NC or sh‐circ‐TNPO3 and co‐transfected with si‐SERPINH1 or control.

We investigated the role of SERPINH1 on the migration of ccRCC cells and determined the functional interplay between circ‐TNPO3 and the IGF2BP2/SERPINH1 axis. We compared the expression of SERPINH1 in ccRCC tissues relative to matched adjacent noncancerous tissues (*n* = 110) and demonstrated that SERPINH1 mRNA expression was significantly increased in ccRCC tissues (Figure 8A). We confirmed successful interference and overexpression of SERPINH1 by transfecting the siRNA and overexpression plasmid of SERPINH1, respectively in Caki‐1 and RCC‐JF cells (Figure S10A,B). In vitro migration assay indicated that the knockdown of SERPINH1 significantly inhibited, whereas its overexpression promoted the migration ability of ccRCC cells (Figure 8B). CCK8, colony formation and EdU assays confirmed that the inhibition of SERPINH1 significantly inhibited, whereas the overexpression of SERPINH1 promoted the proliferation of ccRCC cells (Figure S11A–C). Next, we constructed an abdominal dissemination model in nude mice using Caki‐1 cell line with stably knocked‐down SERPINH1. The results indicated that the inhibition of SERPINH1 obviously decreased the dissemination of abdominal tumour nodules as compared to controls (Figure S12). Therefore, our data suggested that SERPINH1 promoted the proliferation and migration of ccRCC cells. Rescue assays showed that the knockdown of SERPINH1 significantly impaired si‐circ‐TNPO3‐induced increased migration of ccRCC cells (Figure 8C). Additionally, we verified that SERPINH1 upregulated the expression of SNAIL and SLUG, which participate in the EMT process (Figure 8D). Moreover, si‐SERPINH1 significantly rescued sh‐circ‐TNPO3‐induced enhanced expression of SNAIL and SLUG (Figure 8E). In summary, our data suggested that SERPINH1 functions as the target of circ‐TNPO3/IGF2BP2 and SERPINH1‐SNAIL/SLUG axis may be required for circ‐TNPO3‐mediated suppression of ccRCC cell migration.

### 3.9 ESRP1 is probably involved in the biogenesis of circ‐TNPO3 via targeting the flanking intron

**FIGURE 9:**
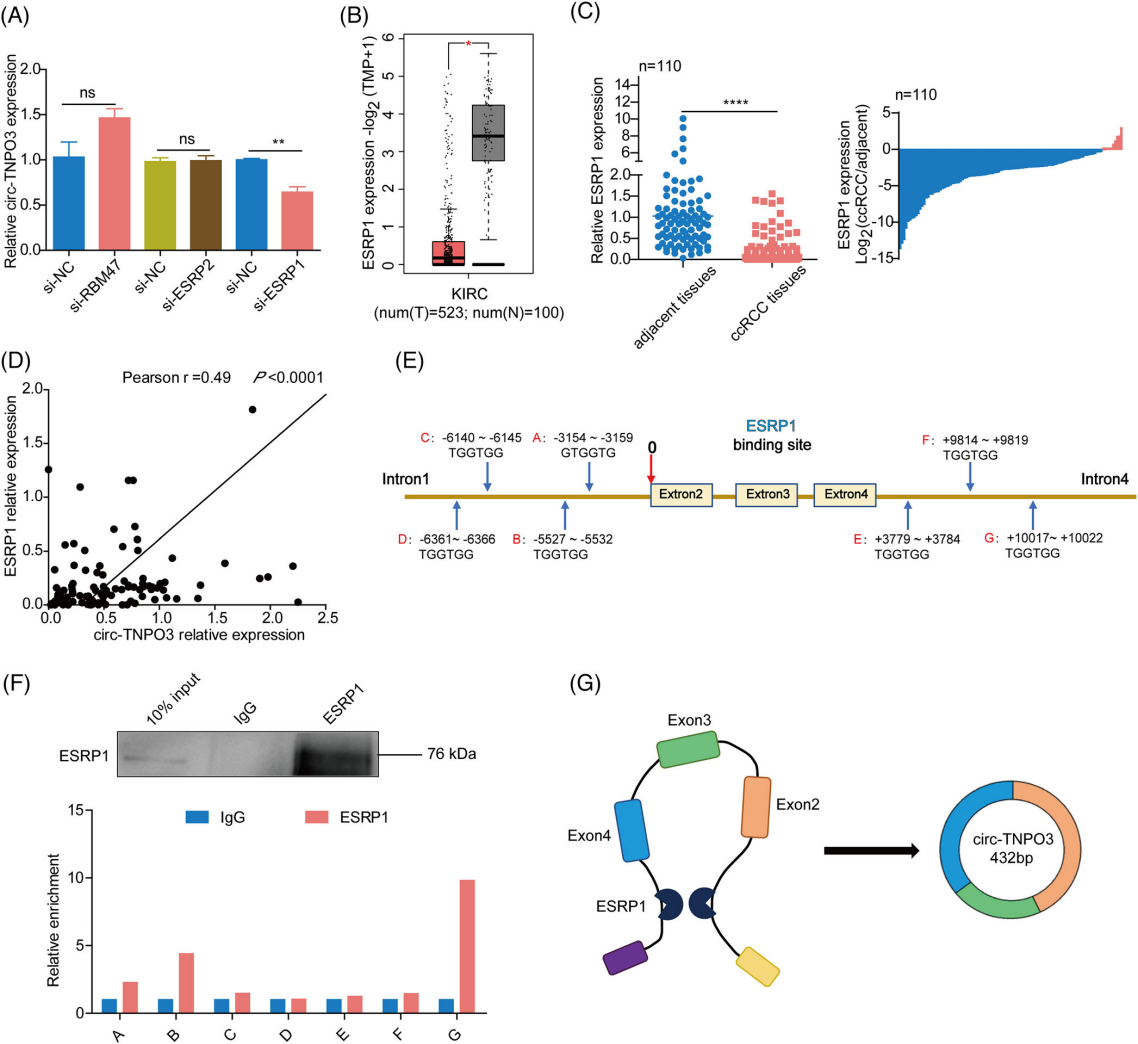
Epithelial splicing regulatory protein 1 (ESRP1) is probably involved in the biogenesis of circ‐transportin‐3 (TNPO3) via targeting the flanking intron. (A) Quantitative real‐time (qRT)‐PCR showed the circ‐TNPO3 expression in Caki‐1 cells transfected with candidate RBPs silencing siRNA or controls. Data were shown as mean ± SD. Student's *t*‐test, *n* = 3. ns, not significant. **p < .01. (B) Analysis of mRNA levels of ESRP1 in clear cell renal cell carcinoma (ccRCC) from TCGA datasets. (C) qRT‐PCR analysed the mRNA levels of ESRP1 in ccRCC tissues. Paired *t*‐test, *n* = 110. ****p < .0001. (D) The correlation between the expression of ESRP1 and circ‐TNPO3 in ccRCC tissues. Pearson test, *n* = 110. *r* = .49, *p* < .0001. (E) Multiple ESRP1 binding sites were observed in the flanking of circ‐TNPO3. (F) The binding of ESRP1 to circ‐TNPO3 flanks was verified by RNA immunoprecipitation (RIP) experiments. (G) schematic diagram showed ESRP1 promotes the biogenesis of circ‐TNPO3.

Given that some splicing factors were able to regulate the biogenesis of circRNAs, we tried to identify which splicing factor regulates the transcription of circ‐TNPO3. Among the circRNAs‐related splicing factors, we analysed their expression in ccRCC tissues compared with adjacent noncancerous based on the TCGA database. We found three splicing factors with differential expression in ccRCC, including ESRP1, ESRP2 and RBM47 as the candidate regulators for circ‐TNPO3 expression. Then we verified the expression of circ‐TNPO3 was evidently reduced when silencing ESRP1 in ccRCC cells, whereas other splicing factors did not show the effect on the expression of circ‐TNPO3 (Figure 9A). Next, we compared the expression of ESRP1 in 110 ccRCC tissues with their matched normal tissues and found the downregulation of ESRP1 in ccRCC tissues, consistent with TCGA data from 523 ccRCC patients and 100 normal tissues (Figure 9B,C). Moreover, we found that the mRNA level of ESRP1 and circ‐TNPO3 was positively correlated in ccRCC (Figure 9D). Splicing factors have been reported to induce circRNA formation by directly binding to the sequences on introns adjacent to the exons which form circRNAs. Interestingly, multiple ESRP1 motifs (GGT‐rich) were observed in the flanking of circ‐TNPO3 (Figure 9E). Subsequently, the RIP assay indicated that ESRP1 mainly binds to two motifs sites (B in intron one and G in intron four) of circ‐TNPO3 flanks (Figure 9F). Taken together, the above data suggest that splicing factor ESRP1 may improve the biogenesis of circ‐TNPO3 via targeting the flanking intron (Figure 9G).

## 4 DISCUSSION

Recently, several circRNAs have been implicated in tumourigenesis and the development of cancer.^9^, ^28^ In ccRCC, various circRNAs regulate different pathological processes, such as cell proliferation, apoptosis, migration and invasion. For example, Xue et al. showed that circ‐AKT3 competitively binds to miR‐296‐3p, resulting in the upregulation of E‐cadherin, thereby suppressing ccRCC metastasis.^29^ Cen et al. confirmed that circSDHC, as an oncogenic factor, can enhance the growth and metastasis of ccRCC by the miR‐127‐3p/CDKN3/E2F1 axis.^30^ In our study, we proved that circ‐TNPO3, a novel tumour suppressor, was downregulated in ccRCC and suppressed the migration and proliferation of ccRCC cells. Circ‐TNPO3 showed a good correlation with some clinicopathological features. The expression of circ‐TNPO3 in patients with distant metastasis and advanced stages (WHO/ISUP grade III and IV, or T2a–T4) was obviously lower relative to those at early stages (WHO/ISUP grade I and II, or T1a–T1b) of ccRCC. The data demonstrated that circ‐TNPO3 is a potential biomarker for diagnosis and prognosis of ccRCC. Interestingly, we previously reported that circ‐TNPO3 suppressed migration by serving as a protein decoy in gastric cancer.^31^ However, another study reports that circ‐TNPO3 plays the oncogenic effects, thus contributing to PTX tolerance through the miR‐1299‐NEK2 axis in ovarian cancer.^32^ Thus, the above evidence suggests that the function of circ‐TNPO3 shows diversity and complexity and may exert versatile roles in various tumours.

To date, the precise mechanism through which circRNAs modulate the expression of target genes is uncertain. Herein, we tried to uncover the interaction between tumour‐associated RBPs and circ‐TNPO3. circRNAs can bind to various proteins and act as protein decoys, sponges, scaffolds or recruiters.^19^, ^33^ Here, we validated that circ‐TNPO3 could bind directly to IGF2BP2, belonging to the IGF2BPs family, which is associated with tumour development. Dysregulation of IGF2BP2 is commonly associated with several human diseases such as cancer, diabetes or insulin resistance.^21^ However, the function and expression of IGF2BP2 are not consistent in different tumours. Many studies report that IGF2BP2 is upregulated and can promote the growth and metastasis of several forms of tumours, such as thyroid carcinoma,^34^ hepatic carcinoma,^35^ pancreatic carcinoma^36^ and colorectal carcinoma.^37^ However, a recent study through a systematic screen for homozygous deletions of 12 human tumours, including RCC, suggests that IGF2BP2 may be a potential tumour suppressor gene.^38^ IGF2BP2 is downregulated in several tumours, including breast‐invasive carcinoma, adrenocortical carcinoma, pheochromocytoma and ccRCC according to TCGA database. In our research, we reported for the first time that IGF2BP2 was downregulated in ccRCC tissues. Functional assays confirmed that IGF2BP2 inhibited the migration of ccRCC cells in vitro. These results indicated IGF2BP2 as a potential tumour suppressor of ccRCC, thus warranting further investigation.

In mammals, the three proteins of the IGF2BPs family share similar structures. IGF2BP2 is composed of two RRMs domains and four KH domains and play regulatory roles in post‐transcriptional processes through the IGF2BP2‐RNA complex.^21^ A growing body of evidence indicates IGF2BP2 can interact with several types of RNAs, including circRNAs. For example, Li et al. showed that circNDUFB2 inhibits NSCLC progression by binding to and destabilizing IGF2BP2, thereby activating anti‐tumour immunity.^39^ Li et al. showed that circCD44 promotes TNBC progression in part by interacting with IGF2BP2.^40^ Ji et al. suggested that circARHGAP12 can directly bind to IGF2BP2, thus improving the stability of FOXM1 mRNA.^41^ It was shown that the KH structural domain of IMP2 can confer sequence‐specific recognition of RNA through its variable loop region.^42^ In addition, the KH domain of IGF2BP2 has been reported to also bind specifically to circNSUN2 and is required for the recruitment of circNSUN2.^43^ In the current research, circ‐TNPO3 bound to both the RRM and KH regions of IGF2BP2 but predominantly bound with the complete KH domain (193–913 aa). However, which region of circ‐TNPO3 is responsible for the binding to IGF2BP2 needs further investigation.

IGF2BP2 modulates tumour progression by serving as a post‐transcriptional regulatory factor for the target mRNAs of MYC,^34^ HMGA2,^44^ MIS12,^45^ SOX2^37^ and FEN1.^35^ We confirmed that SERPINH1 acted as the target of the circ‐TNPO3‐IGF2BP2 axis. IGF2BP2 and circ‐TNPO3 could suppress ccRCC migration by inhibiting the expression of SERPINH1. SERPINH1 belongs to the serpin family and plays the role of a molecular chaperone in collagen biosynthesis.^24^ SERPINH1 is involved in tumour progression, diagnosis and therapy. For instance, Xiong et al. showed that SERPINH1 promotes frontal migration and colonization of cancer cells by enhancing the interaction between cancer cells and platelets.^46^ Zhu et al. demonstrated that SERPINH1 promotes tumour progression by regulating ECM deposition and is involved in poor prognosis of breast cancer.^47^ Herein, we identified that circ‐TNPO3 inhibited the expression of SNAIL and SLUG via SERPINH1. SNAIL and SLUG belong to the Snail family of proteins which have been extensively studied and reported as the master regulator of EMT in breast,^48^ liver,^49^ colorectum^50^ and renal.^51^ In our study, circ‐TNPO3 directly interacted with IGF2BP2 and regulated the downstream SERPINH1‐SNAIL/SLUG axis.

Notably, IGF2BP2 can promote target expression by enhancing mRNA stability. In our study, we found that IGF2BP2 could inhibit the stability of SERPINH1 mRNA. This is the first report on IGF2BP2 destabilizing its target mRNA, thus indicating the complexity of the regulatory role of IGF2BP2. As another member of the IGF2BP family, IGF2BP3 reportedly promotes eIF4E‐mediated translational activation by decreasing the stability of EIF4E‐BP2 mRNA, which in turn enhances the proliferation of tumour cells.^52^ In addition, YTHDF2, a common RNA binding protein, has been reported to inhibit the mRNA stability of several target genes, including MYH7,^53^EGFR,^54^UBXN1,^55^LXRA and HIVEP2.^56^ As a famous m6A reader, IGF2BP2 can enhance mRNA stability through its recognition of RNA N6‐methyladenosine. Recent findings show that IGF2BP2 can regulate the mRNA stability of FEN1,^35^ MYC,^34^ HMGA1^57^ and DANCR^58^ through the m6A modifications. Therefore, we tried to examine whether IGF2BP2 affected the expression of SERPINH1 through the m6A modifications. We performed RIP experiments using m6A antibodies in Caki‐1 cells with IGF2BP2 interference or siRNA control. The results showed that m6A modifications existed in SERPINH1, and m6A‐modified SERPINH1 mRNA was significantly enhanced after IGF2BP2 interference (Figure S13). This suggested that IGF2BP2 may inhibit the expression of SERPINH1 through m6A modification. However, IGF2BP family proteins usually enhance m6A mRNA stability.^59^ Therefore, further investigations are required to elucidate how IGF2BP2 contributes to destabilizing of m6A‐modified SERPINH1 mRNA.

The regulation of circRNAs biogenesis is complex. First, Pol II binds and transcribes pre‐mRNAs that produce circRNAs, turning on the regulation of circRNA biogenesis. Second, *cis*‐ and *trans*‐regulatory factors further regulate the efficiency of circRNA back‐splicing, and these factors include regulatory RBPs, ICSs flanking circle formation exons and core spliceosome components. In addition, circRNA turnover is also closely related to its expression.^60^ Among them, the RBP‐dependent cyclization pattern is an important mechanism of circRNA formation, and common regulatory RBPs include MBI,^12^ QKI,^61^ DHX9^62^ and ESRP1.^63^, ^64^ In our study, we identified that the splicing factor, ESRP1, accelerated the biogenesis of circ‐TNPO3, probably owing to the shared GGT‐rich region of introns 1 and 4 of the circ‐TNPO3 precursor mRNA. Existing studies demonstrate that ESRP1 is involved in the biogenesis of other circRNAs except for circ‐TNPO3. For instance, ESRP1 increases the formation of circUHRF1 by targeting flanking introns in OSCC, thus promoting tumour migration.^63^ In addition, ESRP1 also promotes the biogenesis of circANKS1B^64^ and circBIRC6.^65^ Notable, ESRP1 was significantly downregulated in ccRCC tissues compared with matched normal tissues. Our study provides new clues to elucidate the regulatory mechanism underlying circ‐TNPO3 expression.

## 5 CONCLUSION

**FIGURE 10:**
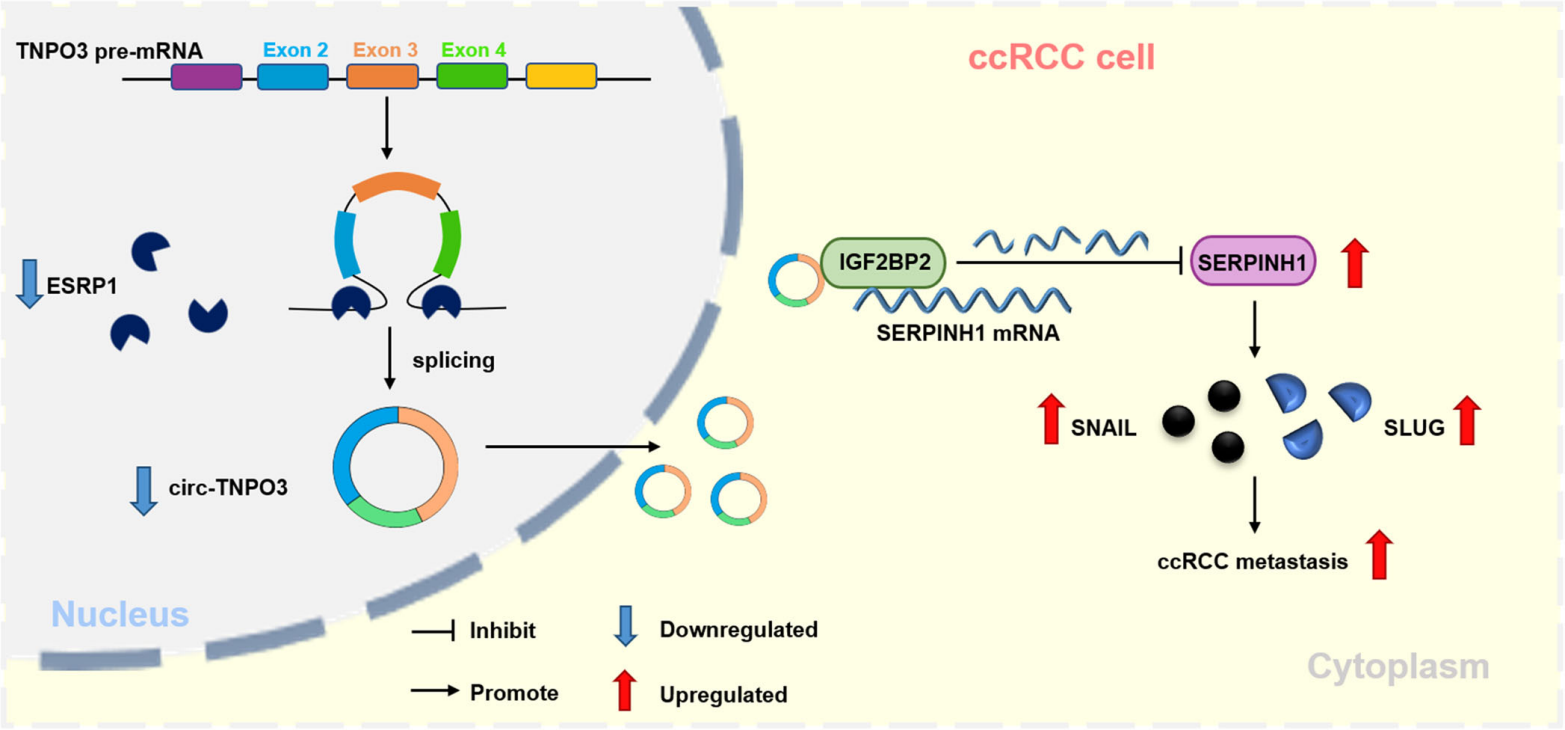
The schematic diagram elucidates the function and mechanism of circ‐transportin‐3 (TNPO3) action in clear cell renal cell carcinoma (ccRCC). Circ‐TNPO3 is downregulated in ccRCC. Circ‐TNPO3 can suppress ccRCC metastasis by directly binding to insulin‐like growth factor 2 mRNA‐binding protein 2 (IGF2BP2) protein and destabilizing serpin family H member 1 (SERPINH1) mRNA, in turn, regulating the SERPINH1‐SNAIL/SLUG axis. Epithelial splicing regulatory protein 1 (ESRP1) can improve the biogenesis of circ‐TNPO3 by targeting the flanking intron.

In summary, we verified the downregulation of circ‐TNPO3 in ccRCC and its negative correlation with distant metastasis, WHO/ISUP grade and tumour T stage. Functionally, circ‐TNPO3 suppressed the proliferation and migration of ccRCC cells in vivo and in vitro. Mechanistically, circ‐TNPO3 directly interacted with IGF2BP2 and downregulated IGF2BP2‐mediated SERPINH1 expression by decreasing the stability of SERPINH1 mRNA. ESRP1 is probably involved in the biogenesis of circ‐TNPO3 by targeting the flanking intron. In the future, circ‐TNPO3 may become a potential target for the diagnosis and treatment of ccRCC (Figure 10).

## CONFLICT OF INTEREST

The authors declare that there is no conflict of interest.

## Supporting information

Supporting Information

Supporting Information

## Data Availability

The analysed data sets generated during the study are available from the corresponding author on reasonable request

## References

[1] Chow WH , Devesa SS , Warren JL , et al. Rising incidence of renal cell cancer in the United States. JAMA. 1999;281(17):1628‐1631. 10235157 10.1001/jama.281.17.1628

[2] Sung H , Ferlay J , Siegel RL , et al. Global cancer statistics 2020: GLOBOCAN estimates of incidence and mortality worldwide for 36 cancers in 185 countries. CA Cancer J Clin. 2021;71(3):209‐249. 33538338 10.3322/caac.21660

[3] Shuch B , Amin A , Armstrong AJ , et al. Understanding pathologic variants of renal cell carcinoma: distilling therapeutic opportunities from biologic complexity. Eur Urol. 2015;67(1):85‐97. 24857407 10.1016/j.eururo.2014.04.029

[4] Ljungberg B , Campbell SC , Choi HY , et al. Corrigendum to “the epidemiology of renal cell carcinoma” [Eur Urol 2011;60:615‐21]. Eur Urol. 2011;60(6):1317. 27989534 10.1016/j.eururo.2011.09.001

[5] Hsieh JJ , Purdue MP , Signoretti S , et al. Renal cell carcinoma. Nat Rev Dis Primers. 2017;3:17009. 28276433 10.1038/nrdp.2017.9 PMC5936048

[6] De Meerleer G , Khoo V , Escudier B , et al. Radiotherapy for renal‐cell carcinoma. Lancet Oncol. 2014;15(4):e170‐e177. 24694640 10.1016/S1470-2045(13)70569-2

[7] Moch H , Srigley J , Delahunt B , et al. Biomarkers in renal cancer. Virchows Arch. 2014;464(3):359‐365. 24487793 10.1007/s00428-014-1546-1

[8] Chen YY , Hu HH , Wang YN , et al. Metabolomics in renal cell carcinoma: from biomarker identification to pathomechanism insights. Arch Biochem Biophys. 2020;695:108623. 33039388 10.1016/j.abb.2020.108623

[9] Memczak S , Jens M , Elefsinioti A , et al. Circular RNAs are a large class of animal RNAs with regulatory potency. Nature. 2013;495(7441):333‐338. 23446348 10.1038/nature11928

[10] Kristensen LS , Andersen MS , Stagsted LVW , et al. The biogenesis, biology and characterization of circular RNAs. Nat Rev Genet. 2019;20(11):675‐691. 31395983 10.1038/s41576-019-0158-7

[11] Han B , Chao J , Yao H . Circular RNA and its mechanisms in disease: from the bench to the clinic. Pharmacol Ther. 2018;187:31‐44. 29406246 10.1016/j.pharmthera.2018.01.010

[12] Ashwal‐Fluss R , Meyer M , Pamudurti NR , et al. circRNA biogenesis competes with pre‐mRNA splicing. Mol Cell. 2014;56(1):55‐66. 25242144 10.1016/j.molcel.2014.08.019

[13] Hansen TB , Jensen TI , Clausen BH , et al. Natural RNA circles function as efficient microRNA sponges. Nature. 2013;495(7441):384‐388. 23446346 10.1038/nature11993

[14] Smid M , Wilting SM , Uhr K , et al. The circular RNome of primary breast cancer. Genome Res. 2019;29(3):356‐366. 30692147 10.1101/gr.238121.118 PMC6396421

[15] Cheng Z , Yu C , Cui S , et al. circTP63 functions as a ceRNA to promote lung squamous cell carcinoma progression by upregulating FOXM1. Nat Commun. 2019;10(1):3200. 31324812 10.1038/s41467-019-11162-4 PMC6642174

[16] Wei Y , Chen X , Liang C , et al. A noncoding regulatory RNAs network driven by Circ‐CDYL acts specifically in the early stages hepatocellular carcinoma. Hepatology (Baltimore, Md). 2020;71(1):130‐147. 10.1002/hep.30795 31148183

[17] Hua JT , Chen S , He HH . Landscape of noncoding RNA in prostate cancer. Trends Genet: TIG. 2019;35(11):840‐851. 31623872 10.1016/j.tig.2019.08.004

[18] Chen Q , Liu T , Bao Y , et al. CircRNA cRAPGEF5 inhibits the growth and metastasis of renal cell carcinoma via the miR‐27a‐3p/TXNIP pathway. Cancer Lett. 2020;469:68‐77. 31629934 10.1016/j.canlet.2019.10.017

[19] Zang J , Lu D , Xu A . The interaction of circRNAs and RNA binding proteins: an important part of circRNA maintenance and function. J Neurosci Res. 2020;98(1):87‐97. 30575990 10.1002/jnr.24356

[20] Bell JL , Wächter K , Mühleck B , et al. Insulin‐like growth factor 2 mRNA‐binding proteins (IGF2BPs): post‐transcriptional drivers of cancer progression?. Cell Mol Life Sci: CMLS. 2013;70(15):2657‐2675. 23069990 10.1007/s00018-012-1186-z PMC3708292

[21] Cao J , Mu Q , Huang H . The roles of insulin‐like growth factor 2 mRNA‐binding protein 2 in cancer and cancer stem cells. Stem Cell Int. 2018;2018:4217259. 10.1155/2018/4217259 PMC5874980 29736175

[22] Ito S , Nagata K . Biology of Hsp47 (Serpin H1), a collagen‐specific molecular chaperone. Semin Cell Dev Biol. 2017;62:142‐151. 27838364 10.1016/j.semcdb.2016.11.005

[23] Kojima T , Miyaishi O , Saga S , et al. The retention of abnormal type I procollagen and correlated expression of HSP 47 in fibroblasts from a patient with lethal osteogenesis imperfecta. J Pathol. 1998;184(2):212‐218. 9602714 10.1002/(SICI)1096-9896(199802)184:2<212::AID-PATH996>3.0.CO;2-Z

[24] Duarte BDP , Bonatto D . The heat shock protein 47 as a potential biomarker and a therapeutic agent in cancer research. J Cancer Res Clin Oncol. 2018;144(12):2319‐2328. 30128672 10.1007/s00432-018-2739-9 PMC11813397

[25] Zhang L , Han B , Wang J , et al. Differential expression profiles and functional analysis of circular RNAs in children with fulminant myocarditis. Epigenomics. 2019;11(10):1129‐1141. 31198064 10.2217/epi-2019-0101

[26] Wu Y , Zhao T , Deng R , et al. A study of differential circRNA and lncRNA expressions in COVID‐19‐infected peripheral blood. Sci Rep. 2021;11(1):7991. 33846375 10.1038/s41598-021-86134-0 PMC8041881

[27] Degrauwe N , Schlumpf TB , Janiszewska M , et al. The RNA binding protein IMP2 preserves glioblastoma stem cells by preventing let‐7 target gene silencing. Cell Rep. 2016;15(8):1634‐1647. 27184842 10.1016/j.celrep.2016.04.086

[28] Yu CY , Kuo HC . The emerging roles and functions of circular RNAs and their generation. J Biomed Sci. 2019;26(1):29. 31027496 10.1186/s12929-019-0523-z PMC6485060

[29] Xue D , Wang H , Chen Y , et al. Circ‐AKT3 inhibits clear cell renal cell carcinoma metastasis via altering miR‐296‐3p/E‐cadherin signals. Mol Cancer. 2019;18(1):151. 31672157 10.1186/s12943-019-1072-5 PMC6824104

[30] Cen J , Liang Y , Huang Y , et al. Circular RNA circSDHC serves as a sponge for miR‐127‐3p to promote the proliferation and metastasis of renal cell carcinoma via the CDKN3/E2F1 axis. Mol Cancer. 2021;20(1):19. 33468140 10.1186/s12943-021-01314-w PMC7816303

[31] Yu T , Ran L , Zhao H , et al. Circular RNA circ‐TNPO3 suppresses metastasis of GC by acting as a protein decoy for IGF2BP3 to regulate the expression of MYC and SNAIL. Mol Ther Nucleic Acids. 2021;26:649‐664. 34703650 10.1016/j.omtn.2021.08.029 PMC8516998

[32] Xia B , Zhao Z , Wu Y , et al. Circular RNA circTNPO3 regulates paclitaxel resistance of ovarian cancer cells by miR‐1299/NEK2 signaling pathway. Mol Ther Nucleic Acids. 2020;21:780‐791. 32791450 10.1016/j.omtn.2020.06.002 PMC7419276

[33] Huang A , Zheng H , Wu Z , et al. Circular RNA‐protein interactions: functions, mechanisms, and identification. Theranostics. 2020;10(8):3503‐3517. 32206104 10.7150/thno.42174 PMC7069073

[34] Ye M , Dong S , Hou H , et al. Oncogenic role of long noncoding RNAMALAT1 in thyroid cancer progression through regulation of the miR‐204/IGF2BP2/m6A‐MYC signaling. Mol Ther Nucleic Acids. 2021;23:1‐12. 33312756 10.1016/j.omtn.2020.09.023 PMC7711188

[35] Pu J , Wang J , Qin Z , et al. IGF2BP2 promotes liver cancer growth through an m6A‐FEN1‐dependent mechanism. Front Oncol. 2020;10:578816. 33224879 10.3389/fonc.2020.578816 PMC7667992

[36] Xu X , Yu Y , Zong K , et al. Up‐regulation of IGF2BP2 by multiple mechanisms in pancreatic cancer promotes cancer proliferation by activating the PI3K/Akt signaling pathway. J Exp Clin Cancer Res. 2019;38(1):497. 31852504 10.1186/s13046-019-1470-y PMC6921559

[37] Li T , Hu PS , Zuo Z , et al. METTL3 facilitates tumor progression via an m(6)A‐IGF2BP2‐dependent mechanism in colorectal carcinoma. Mol Cancer. 2019;18(1):112. 31230592 10.1186/s12943-019-1038-7 PMC6589893

[38] Cheng J , Demeulemeester J , Wedge DC , et al. Pan‐cancer analysis of homozygous deletions in primary tumours uncovers rare tumour suppressors. Nat Commun. 2017;8(1):1221. 29089486 10.1038/s41467-017-01355-0 PMC5663922

[39] Li B , Zhu L , Lu C , et al. circNDUFB2 inhibits non‐small cell lung cancer progression via destabilizing IGF2BPs and activating anti‐tumor immunity. Nat Commun. 2021;12(1):295. 33436560 10.1038/s41467-020-20527-z PMC7804955

[40] Li J , Gao X , Zhang Z , et al. CircCD44 plays oncogenic roles in triple‐negative breast cancer by modulating the miR‐502‐5p/KRAS and IGF2BP2/Myc axes. Mol Cancer. 2021;20(1):138. 34696797 10.1186/s12943-021-01444-1 PMC8543802

[41] Ji F , Lu Y , Chen S , et al. IGF2BP2‐modified circular RNA circARHGAP12 promotes cervical cancer progression by interacting m(6)A/FOXM1 manner. Cell Death Discovery. 2021;7(1):215. 34392306 10.1038/s41420-021-00595-w PMC8364552

[42] Biswas J , Patel VL , Bhaskar V , et al. The structural basis for RNA selectivity by the IMP family of RNA‐binding proteins. Nat Commun. 2019;10(1):4440. 31570709 10.1038/s41467-019-12193-7 PMC6768852

[43] Chen RX , Chen X , Xia LP , et al. N(6)‐methyladenosine modification of circNSUN2 facilitates cytoplasmic export and stabilizes HMGA2 to promote colorectal liver metastasis. Nat Commun. 2019;10(1):4695. 31619685 10.1038/s41467-019-12651-2 PMC6795808

[44] Chen RX , Chen X , Xia LP , et al. N(6)‐methyladenosine modification of circNSUN2 facilitates cytoplasmic export and stabilizes HMGA2 to promote colorectal liver metastasis. Nat Commun. 2019;10(1):4695. 31619685 10.1038/s41467-019-12651-2 PMC6795808

[45] Wu Z , Shi Y , Lu M , et al. METTL3 counteracts premature aging via m6A‐dependent stabilization of MIS12 mRNA. Nucleic Acids Res. 2020;48(19):11083‐11096. 33035345 10.1093/nar/gkaa816 PMC7641765

[46] Xiong G , Chen J , Zhang G , et al. Hsp47 promotes cancer metastasis by enhancing collagen‐dependent cancer cell‐platelet interaction. Proc Natl Acad Sci USA. 2020;117(7):3748‐3758. 32015106 10.1073/pnas.1911951117 PMC7035603

[47] Zhu J , Xiong G , Fu H , et al. Chaperone Hsp47 drives malignant growth and invasion by modulating an ECM gene network. Cancer Res. 2015;75(8):1580‐1591. 25744716 10.1158/0008-5472.CAN-14-1027 PMC4401637

[48] Lu L , Chen Z , Lin X , et al. Inhibition of BRD4 suppresses the malignancy of breast cancer cells via regulation of Snail. Cell Death Differ. 2020;27(1):255‐268. 31114028 10.1038/s41418-019-0353-2 PMC7205888

[49] Xu H , Wang H , Zhao W , et al. SUMO1 modification of methyltransferase‐like 3 promotes tumor progression via regulating Snail mRNA homeostasis in hepatocellular carcinoma. Theranostics. 2020;10(13):5671‐5686. 32483411 10.7150/thno.42539 PMC7254988

[50] Zhou Y , Lu L , Jiang G , et al. Targeting CDK7 increases the stability of Snail to promote the dissemination of colorectal cancer. Cell Death Differ. 2019;26(8):1442‐1452. 30451989 10.1038/s41418-018-0222-4 PMC6748077

[51] Nam H , Kundu A , Brinkley GJ , et al. PGC1α suppresses kidney cancer progression by inhibiting collagen‐induced SNAIL expression. Matrix Biol: J Int Soc Matrix Biol. 2020;89:43‐58. 10.1016/j.matbio.2020.01.001 PMC7712461 31982456

[52] Mizutani R , Imamachi N , Suzuki Y , et al. Oncofetal protein IGF2BP3 facilitates the activity of proto‐oncogene protein eIF4E through the destabilization of EIF4E‐BP2 mRNA. Oncogene. 2016;35(27):3495‐3502. 26522719 10.1038/onc.2015.410

[53] Xu H , Wang Z , Chen M , et al. YTHDF2 alleviates cardiac hypertrophy via regulating Myh7 mRNA decoy. Cell Biosci. 2021;11(1):132. 34266473 10.1186/s13578-021-00649-7 PMC8281596

[54] Zhong L , Liao D , Zhang M , et al. YTHDF2 suppresses cell proliferation and growth via destabilizing the EGFR mRNA in hepatocellular carcinoma. Cancer Lett. 2019;442:252‐261. 30423408 10.1016/j.canlet.2018.11.006

[55] Chai RC , Chang YZ , Chang X , et al. YTHDF2 facilitates UBXN1 mRNA decay by recognizing METTL3‐mediated m(6)A modification to activate NF‐κB and promote the malignant progression of glioma. J Hematol Oncol. 2021;14(1):109. 34246306 10.1186/s13045-021-01124-z PMC8272379

[56] Fang R , Chen X , Zhang S , et al. EGFR/SRC/ERK‐stabilized YTHDF2 promotes cholesterol dysregulation and invasive growth of glioblastoma. Nat Commun. 2021;12(1):177. 33420027 10.1038/s41467-020-20379-7 PMC7794382

[57] Hou P , Meng S , Li M , et al. LINC00460/DHX9/IGF2BP2 complex promotes colorectal cancer proliferation and metastasis by mediating HMGA1 mRNA stability depending on m6A modification. J Exp Clin Cancer Res. 2021;40(1):52. 33526059 10.1186/s13046-021-01857-2 PMC7851923

[58] Hu X , Peng WX , Zhou H , et al. IGF2BP2 regulates DANCR by serving as an N6‐methyladenosine reader. Cell Death Differ. 2020;27(6):1782‐1794. 31804607 10.1038/s41418-019-0461-z PMC7244758

[59] Huang H , Weng H , Sun W , et al. Recognition of RNA N(6)‐methyladenosine by IGF2BP proteins enhances mRNA stability and translation. Nat Cell Biol. 2018;20(3):285‐295. 29476152 10.1038/s41556-018-0045-z PMC5826585

[60] Li X , Yang L , Chen LL . The biogenesis, functions, and challenges of circular RNAs. Mol Cell. 2018;71(3):428‐442. 30057200 10.1016/j.molcel.2018.06.034

[61] Conn SJ , Pillman KA , Toubia J , et al. The RNA binding protein quaking regulates formation of circRNAs. Cell. 2015;160(6):1125‐1134. 25768908 10.1016/j.cell.2015.02.014

[62] Hong H , An O , Chan THM , et al. Bidirectional regulation of adenosine‐to‐inosine (A‐to‐I) RNA editing by DEAH box helicase 9 (DHX9) in cancer. Nucleic Acids Res. 2018;46(15):7953‐7969. 29796672 10.1093/nar/gky396 PMC6125626

[63] Zhao W , Cui Y , Liu L , et al. Splicing factor derived circular RNA circUHRF1 accelerates oral squamous cell carcinoma tumorigenesis via feedback loop. Cell Death Differ. 2020;27(3):919‐933. 31570856 10.1038/s41418-019-0423-5 PMC7206121

[64] Zeng K , He B , Yang BB , et al. The pro‐metastasis effect of circANKS1B in breast cancer. Mol Cancer. 2018;17(1):160. 30454010 10.1186/s12943-018-0914-x PMC6240936

[65] Yu CY , Li TC , Wu YY , et al. The circular RNA circBIRC6 participates in the molecular circuitry controlling human pluripotency. Nat Commun. 2017;8(1):1149. 29074849 10.1038/s41467-017-01216-w PMC5658440

