# Supplementary material for: Circular RNA circ‐TNPO3 inhibits clear cell renal cell carcinoma metastasis by binding to IGF2BP2 and destabilizing SERPINH1 mRNA
Source: Clin Transl Med. 2022 Jul 25;12(7):e994. doi: 10.1002/ctm2.994 (PMC9309750; doi:10.1002/ctm2.994)
Supplement: Supplementary file 1 — Supporting Information [file CTM2-12-e994-s002.docx]

1. **Supplementary materials and methods**

**1.1 Cell culture**

Human ccRCC cell lines (Caki-1, RCC-JF, 786-O) were purchased from MeisenCTCC (Zhejiang, China). All ccRCC cell lines were grown in high glucose DMEM or RPMI-1640 (hyclone, Utah, USA) supplemented with 10% fetal bovine serum (FBS, PAN BERATECH, Germany). Each cell line was cultured as a monolayer at 37℃, 5% carbon dioxide in a humidified atmosphere. The medium was replaced every two days.

**1.2 RNase R and** **actinomycin D assay**

For RNase R treatment, following the manufacturer’s protocol, total RNA (5 μg) was incubated with or without RNase R (3 U/μg) at 37 °C for 15 min. Finally, changes in the expression of circ-TNPO3 and linear TNPO3 were detected by qRT-PCR. For the actinomycin D assay, 10 μg/ml actinomycin D (Genview, Beijing, China) or DMSO was added to ccRCC cells in 12-well plates, and RNA was extracted at the indicated time points, reverse transcribed, and finally, qRT-PCR was performed to detect the stability of the linear RNA and circRNA.

**1.3 Wound healing and Transwell assay**

In the wound healing experiment, first, cells were grown in 6-well plates and subsequently, the monolayer was gently scraped off using a 20 μL pipette tip when the cell confluency reached approximately 80%. Subsequently, the wells were washed twice with phosphate-buffered saline (PBS) and a fresh medium containing no serum was added. Images were acquired at 0h, 6h, 12h, and 24h using an optical microscope. For the Transwell assay, 1×10^5^ cells (without FBS) were inoculated into the upper chamber of 8 μm chambers (Corning, MA, USA). Then, 500 μL of medium containing 10% FBS was added to the lower chamber. After 24h of incubation, the non-migrating cells in the upper chamber were gently wiped, and the migrating cells at the bottom of the chamber were fixed with 4% paraformaldehyde for 10 min, followed by staining with the crystalline violet solution for 8 min. The pictures were obtained and the cells were counted under a microscope.

**1.4 Western blot assay**

Total proteins were extracted from ccRCC tissues and cells using RIPA lysis buffer (Beyotime, Shanghai, China) and denatured in a 100 ℃ metal bath for 10 min. Based on their sizes, proteins were separated by SDS-PAGE and transferred onto PVDF membranes. The transferred proteins were blocked using 5% skimmed milk for 1 h and incubated overnight at 4℃ with an appropriate dilution of the primary antibody. The antibodies used in this study were as follows: IGF2BP2 (1:1000, Millipore, MA, USA), SERPINH1 (1:1000, SAB, Maryland, USA), ESRP1 (1:1000, Proteintech, Wuhan, China), FLAG (1:1000, CST, Boston, USA), SNAIL (1:1000, CST, Boston, USA), SLUG (1:1000, CST, Boston, USA), E-cadherin (1:1000, CST, Boston, USA), N-cadherin (1:1000, CST, Boston, USA), Vimentin (1:1000, CST, Boston, USA), GAPDH (1:1000, Beyotime, Shanghai, China), and β-actin (1:1000, Beyotime, Shanghai, China). Signals were analyzed using the SuperSignal West Dura Extended Duration Substrate Kit (Thermo Fisher Scientific, Inc., Waltham, MA, USA).

**1.5 Proliferation assay for cancer cells**

The proliferation ability of ccRCC cells was tested via CCK-8, colony formation, and EdU assays. In the CCK-8 experiment, the transfected ccRCC cells were inoculated into 96-well plates at 3000 cells/well density, and the CCK8 reagent (biosharp, Anhui, China) was added at 24h, 48h, 72h, 96h, and 120h; the corresponding absorbance values were detected using a microplate reader (Thermo Scientific Varioskan LUX, Waltham, MA, USA) after incubation for 2h. In the colony formation experiment, transfected ccRCC cells were seeded in 6-well plates at 1000 cells/well density and cultured for 2-3 weeks. Subsequently, the cells were fixed with 4% paraformaldehyde for 10 min and stained with the crystalline violet solution for 8 min. In the EdU assay, the transfected ccRCC cells were cultured in 48-well plates at 20,000 cells/well density and incubated for 24h, following which they were incubated with the EdU reagent for 2 to 4 hours. The fluorescent signal was obtained using EdU Apollo DNA in vitro kit (Ribobio, Guangzhou, China) and detected by fluorescence microscopy.

**1.6 Animal experiments**

The animals used in this study were 4–5-week-old male BALB/c nude mice. To establish subcutaneous xenograft models, 786-O cells (sh-circ-TNPO3 or sh-NC) were inoculated subcutaneously at the back of the nude mice at a density of 1×10^6^ cells/animal. The tumor size was measured every 2 days and the tumor volume was estimated according to the following formula: volume = length × width^2^ × 0.5. After two weeks, these nude mice were sacrificed, and the tumors were isolated from their back and weighed. To construct an abdominal dissemination model in nude mice, Caki-1 cells (sh-circ-TNPO3 or sh-NC) were injected into the abdominal cavity of nude mice at a cell density of 2×10^6^ cells/mouse. In vivo imaging was performed after the fourth week of injection to observe abdominal tumor dissemination in nude mice. To construct a lung metastasis model in nude mice, sh-circ-TNPO3 or sh-NC 786-O cells were injected into the tail vein at a density of 2×10^6^ cells/animal. The mice were sacrificed in the sixth week after injection, and the lung tissues were stained with hematoxylin and eosin (HE) to detect the occurrence of lung metastasis. All animal experiments in this study were approved by the Animal Management Committee of Chongqing Medical University.

**1.7 Dual-luciferase reporter assay**

The dual-luciferase reporter vector pMIR-REPORT-circ-TNPO3 was synthesized by Sangon Biotech (Shanghai, China), and mimics of candidate microRNAs were synthesized by GenePharma (Shanghai, China). The mimics and pMIR-REPORT-circ-TNPO3 (or control vector) were co-transfected into HEK-293T cells. Finally, the fluorescence values were detected using the Dual-Luciferase Reporter System Kit. (Promega, MI, USA)

1. **Supplementary Figures**


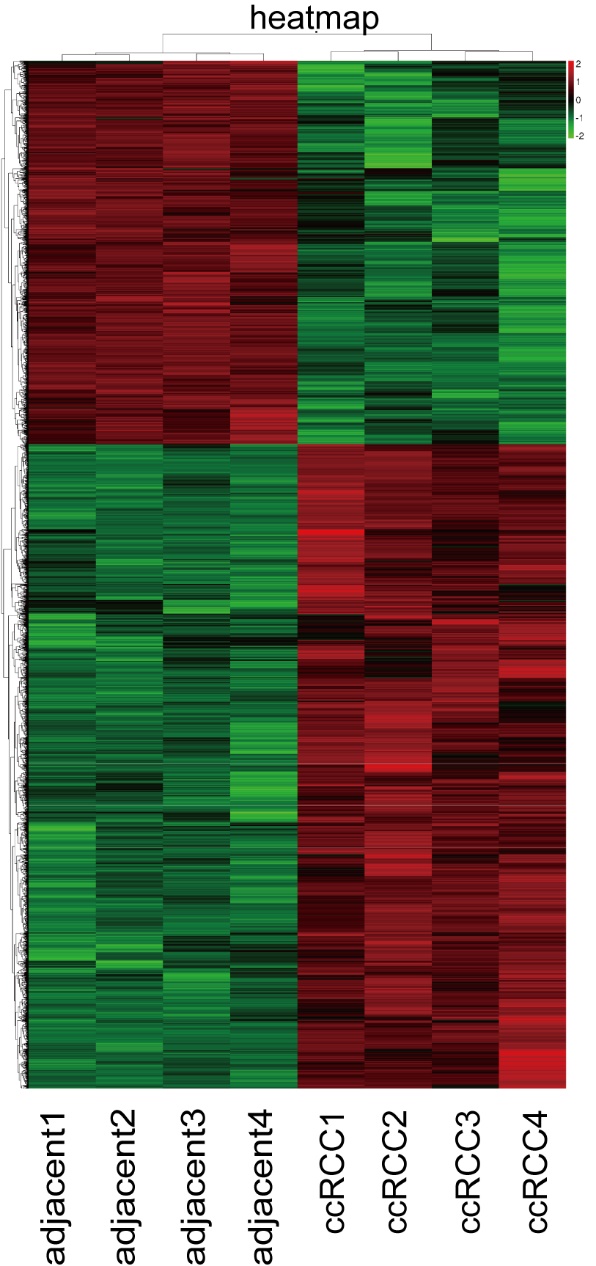


**Figure S1. Screening and identification of differentially expressed circRNA in ccRCC tissues.** Clustered heat map of the identified circRNA signature with 2210 upregulated and 1539 downregulated circRNAs in ccRCC tissues (│log_2_(FC)│≥ 1 and *P* < 0.05).


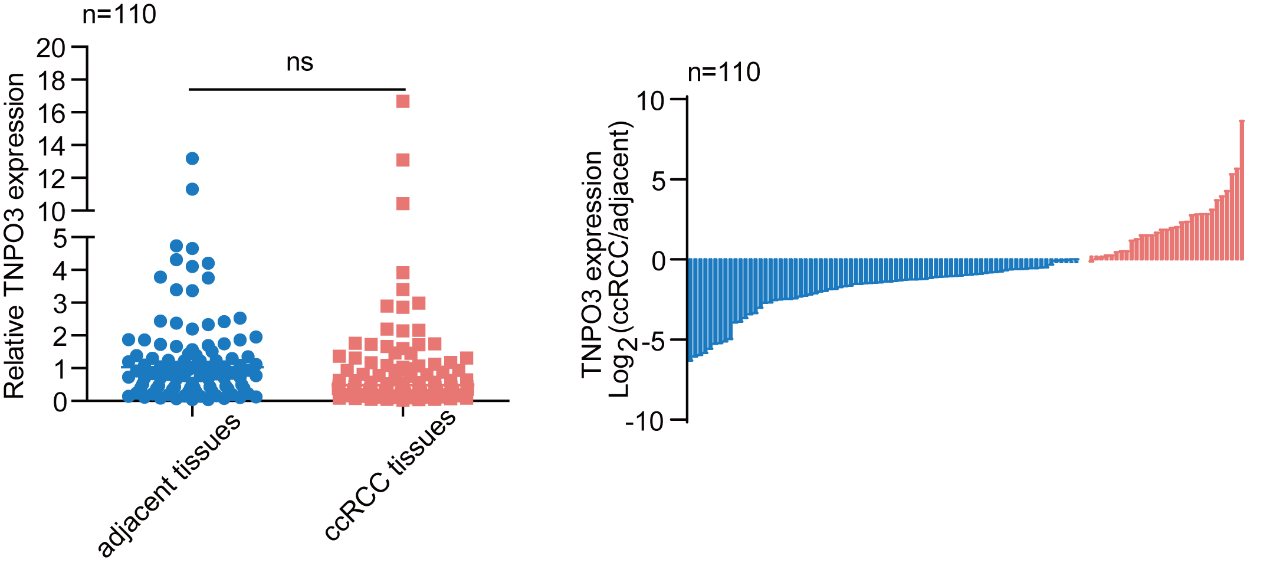


**Figure S2. The mRNA expression levels of TNPO3 in ccRCC.** qRT-PCR analyzed the mRNA levels of parent TNPO3 in ccRCC tissues (n=110). Paired t‐test, n = 110. ns, not significant.


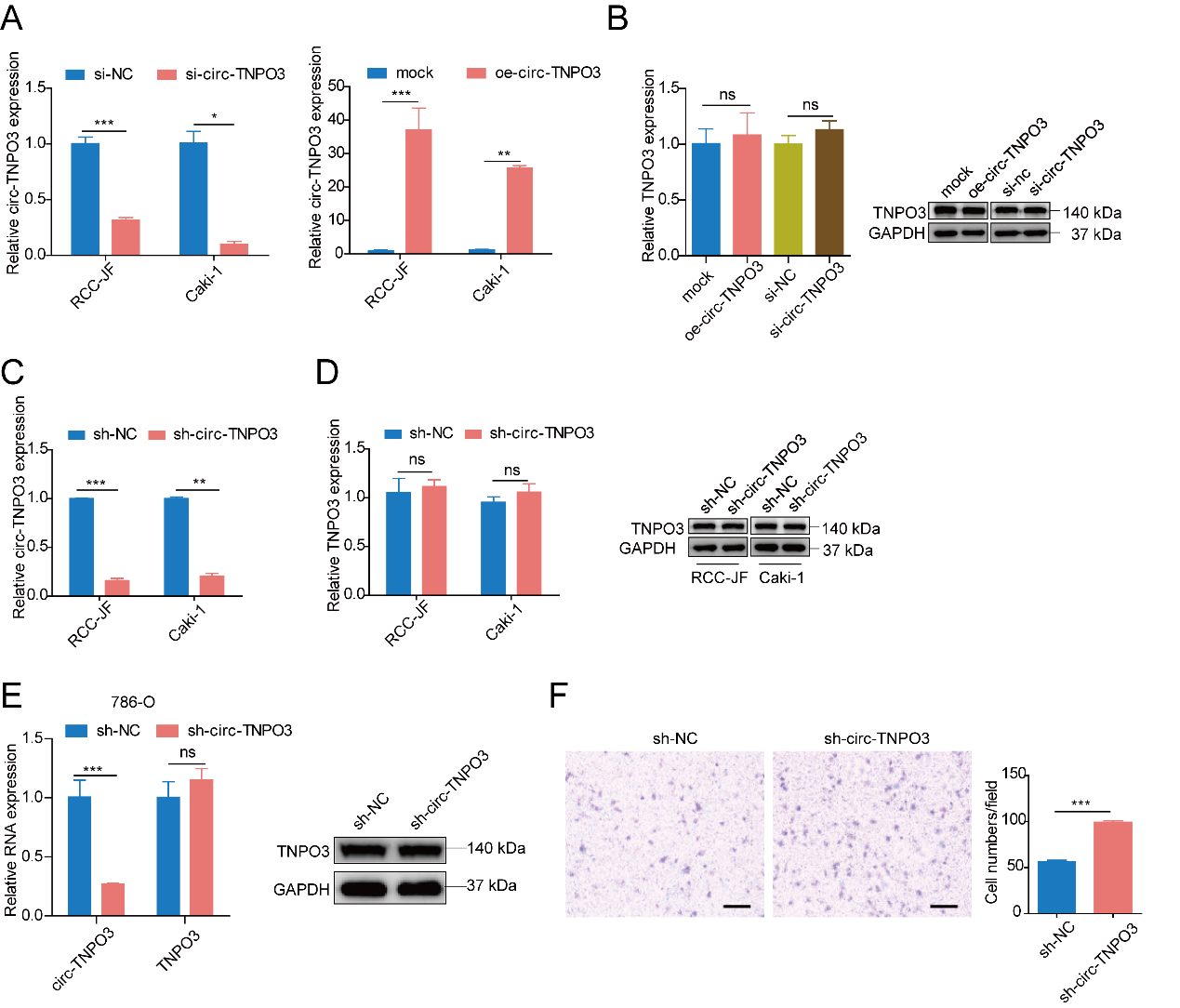


**Figure S3. The efficiency of silencing or overexpression of circ-TNPO3.** (A) The efficiency of silencing or overexpression of circ-TNPO3 in Caki-1 and RCC-JF cells was detected by qRT-PCR. (B) qRT-PCR (left) and Western blot (right) assays showed that knockdown or overexpression of circ-TNPO3 had no influence on liner TNPO3 mRNA and protein levels. (C) The mRNA level of circ-TNPO3 in sh-circ-TNPO3 RCC-JF and Caki-1 cells. (D) qRT-PCR (left) and Western blot (right) assays showing that stably knockdown the expression of circ-TNPO3 had no influence on liner TNPO3 mRNA and protein levels. (E) The mRNA (left) and protein (right) levels of circ-TNPO3 or linear TNPO3 in the sh-circ-TNPO3-786-O cells. (F) The migration ability of sh-circ-TNPO3-786-O cells was tested; scale bars, 50 μm. Data were shown as mean ± SD. Student's t‐test, n = 3. ns, not significant, * *P* < 0.05, ** *P* < 0.01, *** *P* < 0.001.


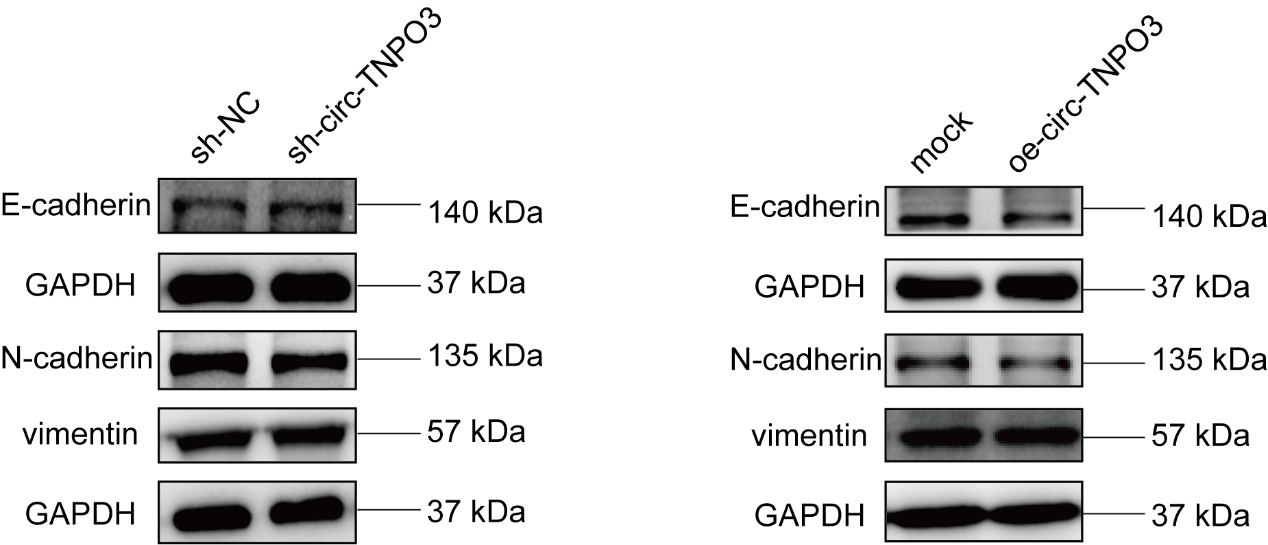


**Figure S4. circ-TNPO3 had no influence on the expression of other EMT-related proteins.** Western blot assay showing that circ-TNPO3 had no significant effect on the expression of E-cadherin, N-cadherin, and vimentin.


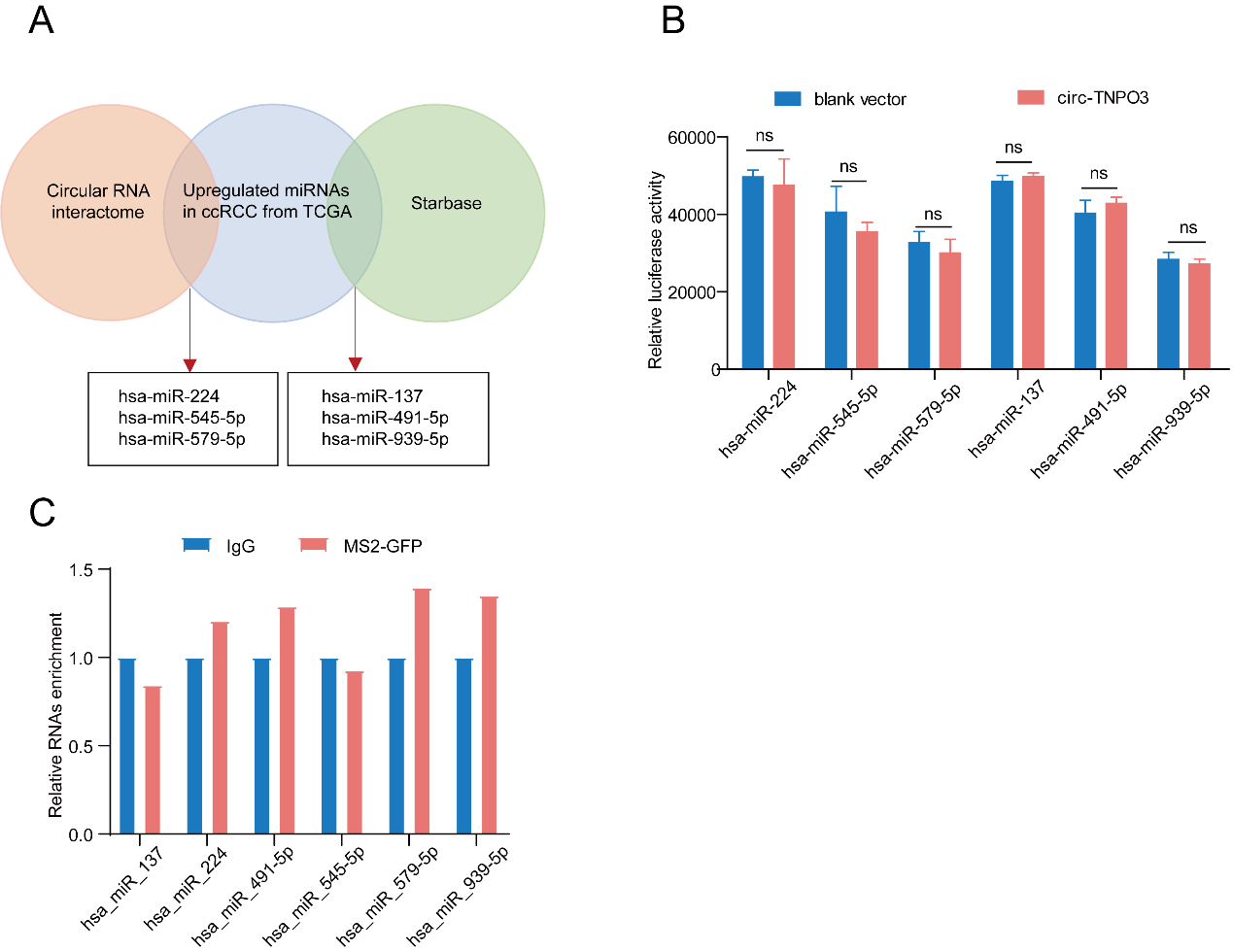


**Figure S5. Circ-TNPO3 may not serve as a miRNA sponge.** (A) Prediction of miRNAs that may bind to circ-TNPO3 by circular RNA interactome, Starbase, and miRNAs expression analysis from TCGA. (B-C) Dual-luciferase reporter assay and RNA pull-down assay were performed to determine the binding of circ-TNPO3 and miRNA candidates. Data were shown as mean ± SD. Student's t‐test, n = 3. ns, not significant.


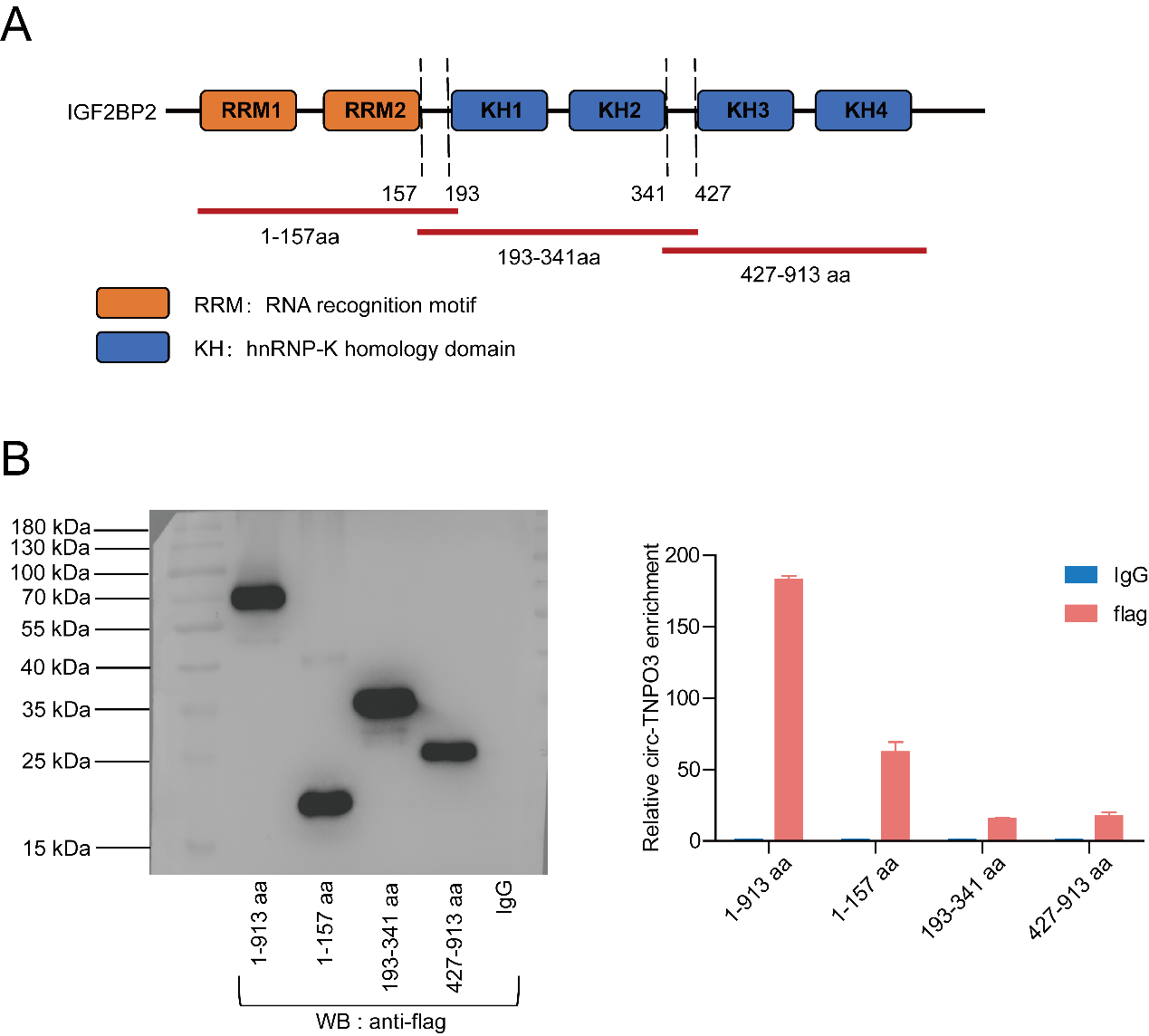


**Figure S6. Validation of the binding site of circ-TNPO3 on IGF2BP2.** (A) Structural diagram of IGF2BP2 protein and three truncated IGF2BP2 variants. (B) The full-length or truncated mutant of IGF2BP2 variants was pulled down by anti-flag which was verified by western blot. The expression of enriched circ-TNPO3 in various groups was tested by qRT-PCR.


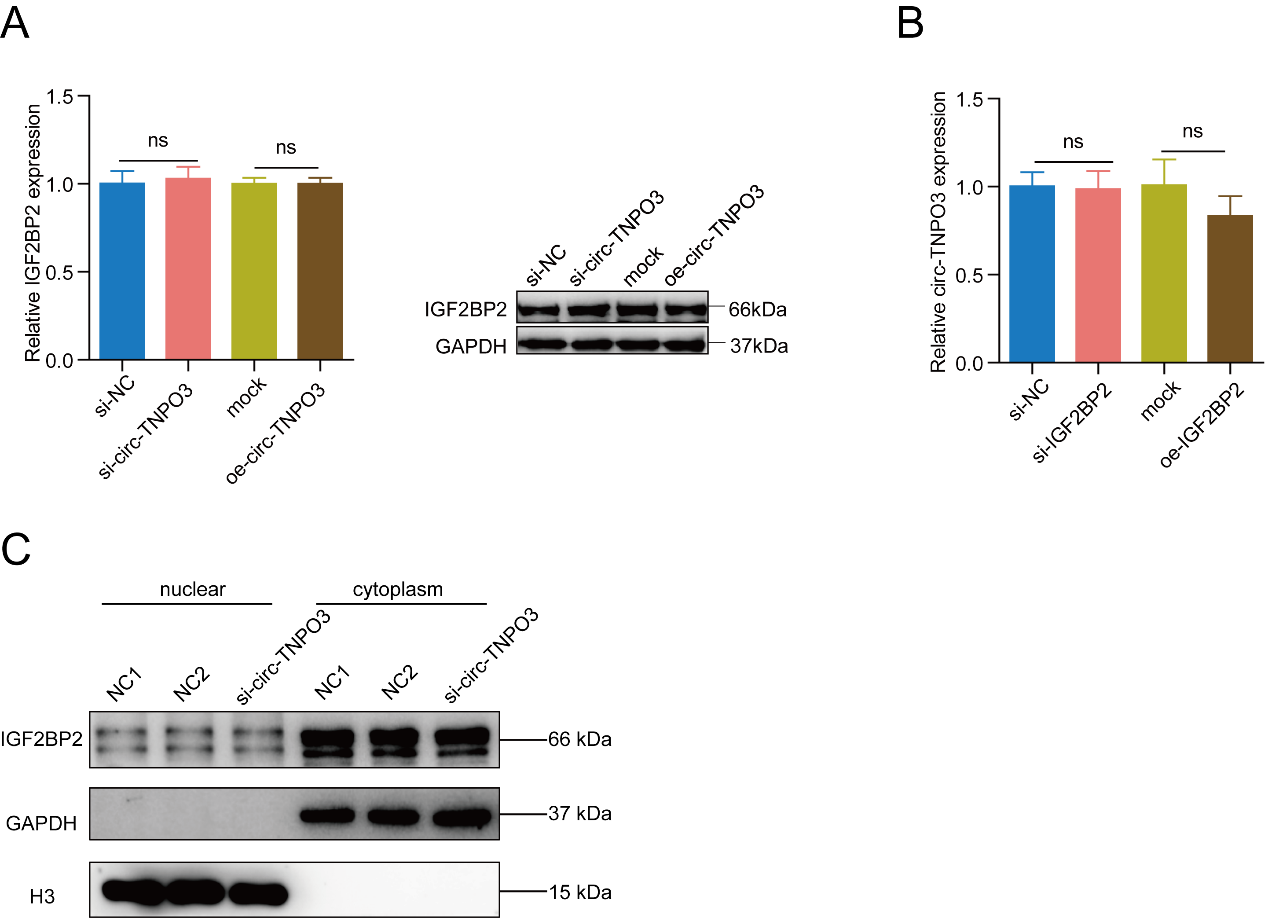


**Figure S7. Analysis of the mutual effects of circ-TNPO3 and IGF2BP2.** (A) qRT-PCR analysis (left) and Western blot assays (right) showed that knockdown or overexpression of circ-TNPO3 had no influence on IGF2BP2 mRNA and protein levels. Data were shown as mean ± SD. Student's t‐test, n = 3. ns, not significant. (B) The qRT-PCR analysis identified that inhibition or overexpression of IGF2BP2 had no influence on the circ-TNPO3 level. Data were shown as mean ± SD. Student's t‐test, n = 3. ns, not significant. (C) Nucleoplasmic separation assay verified the effect of circ-TNPO3 on IGF2BP2 subcellular localization.


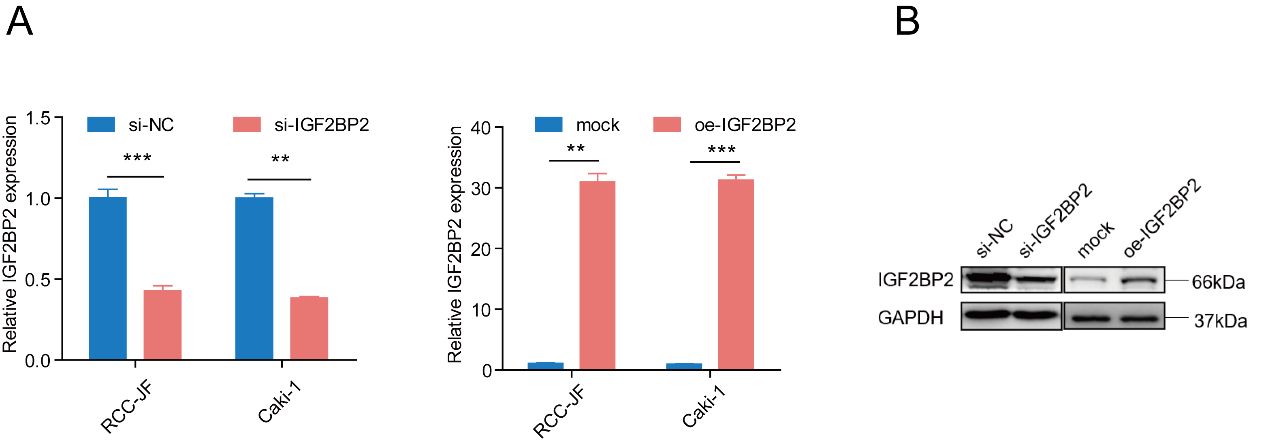


**Figure S8. The efficiency of silencing or overexpression of IGF2BP2**. (A) The mRNA level of IGF2BP2 in RCC-JF and Caki-1 cells transfected with si-IGF2BP2 or oe-IGF2BP2 vector. (B) The protein level of IGF2BP2 transfected with si-IGF2BP2 or oe-IGF2BP2 vector. Data were shown as mean ± SD. Student's t‐test, n = 3. ** *P* < 0.01, *** *P* < 0.001.


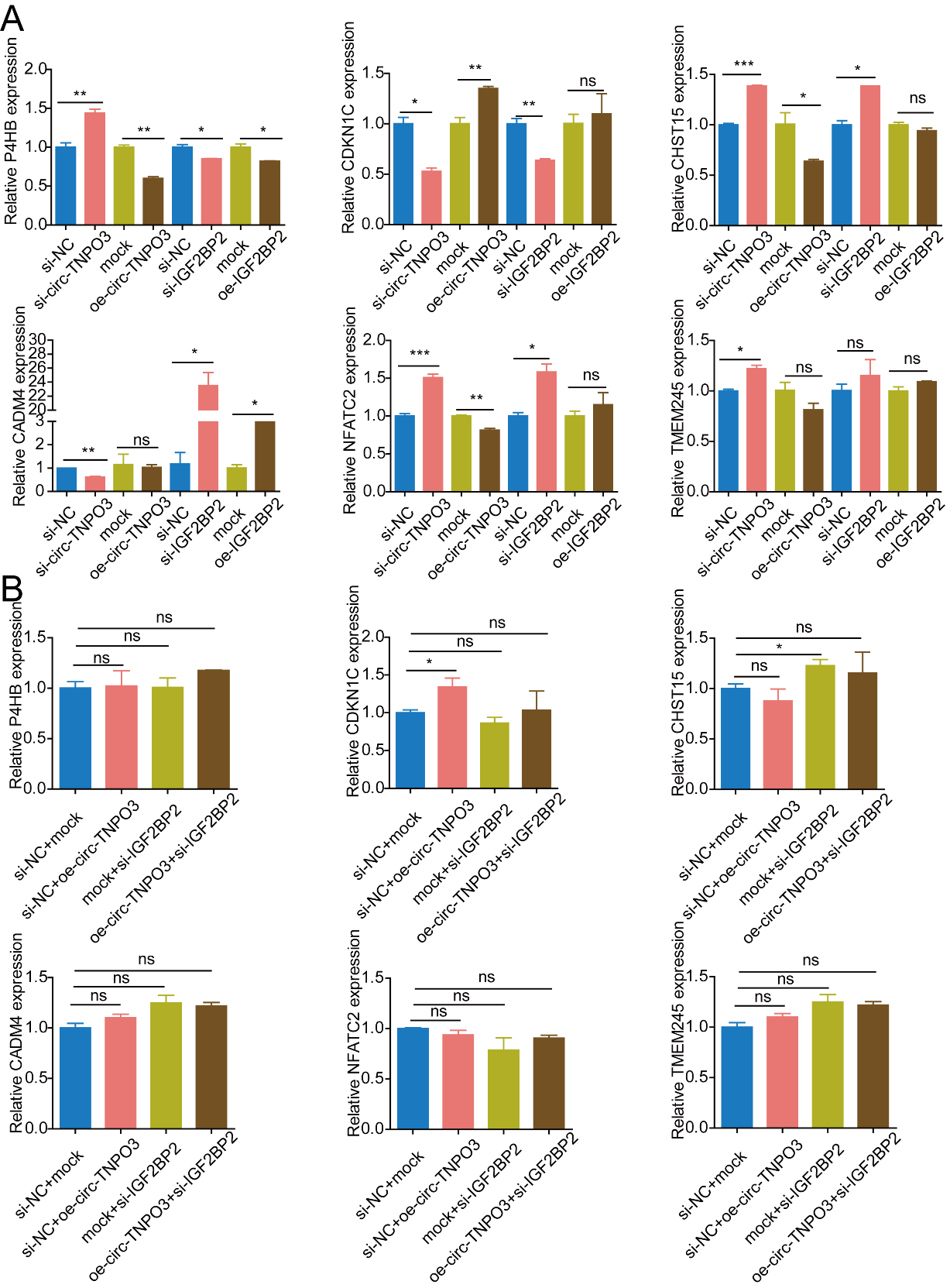


**Figure S9. Identification of candidate targets downstream of the circ-TNPO3-IGF2BP2 axis.** (A) The mRNA levels of candidate targets in Caki-1 cells after interference or overexpression of circ-TNPO3 or IGF2BP2. (B) The mRNA levels of candidate targets in Caki-1 cells transfected with oe-circ-TNPO3, si-IGF2BP2, or oe-circ-TNPO3+ si-IGF2BP2. Data were shown as mean ± SD. Student's t‐test, n = 3. ns, not significant. * *P* < 0.05, ** *P* < 0.01, *** *P* < 0.001.


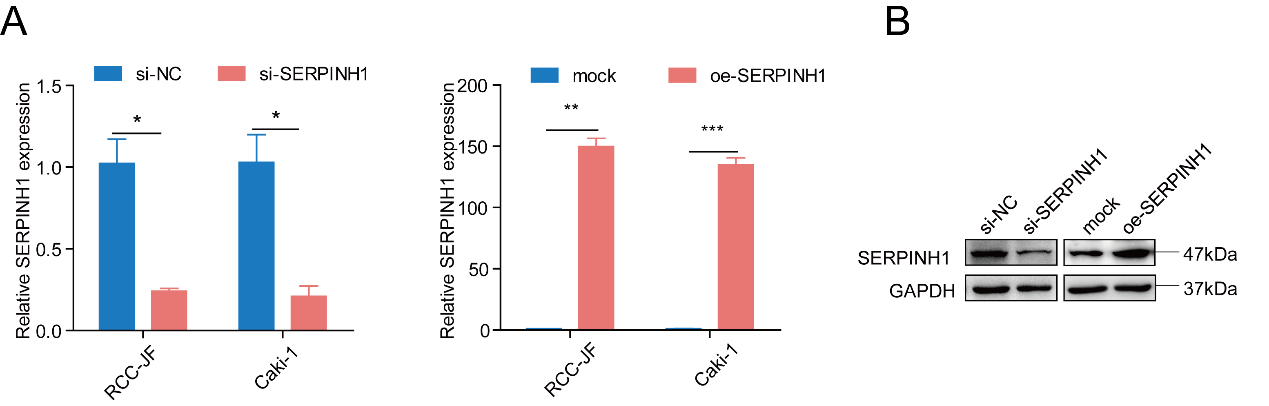


**Figure S10. The efficiency of silencing or overexpression of SERPINH1**. (A) The mRNA level of SERPINH1 in RCC-JF and Caki-1 cells transfected with si-SERPINH1 or oe-SERPINH1 vector. (B) The protein level of SERPINH1 was transfected with the si-SERPINH1 or oe-SERPINH1 vector. Data were shown as mean ± SD. Student's t‐test, n = 3. * *P* < 0.05, ** *P* < 0.01, *** *P* < 0.001.


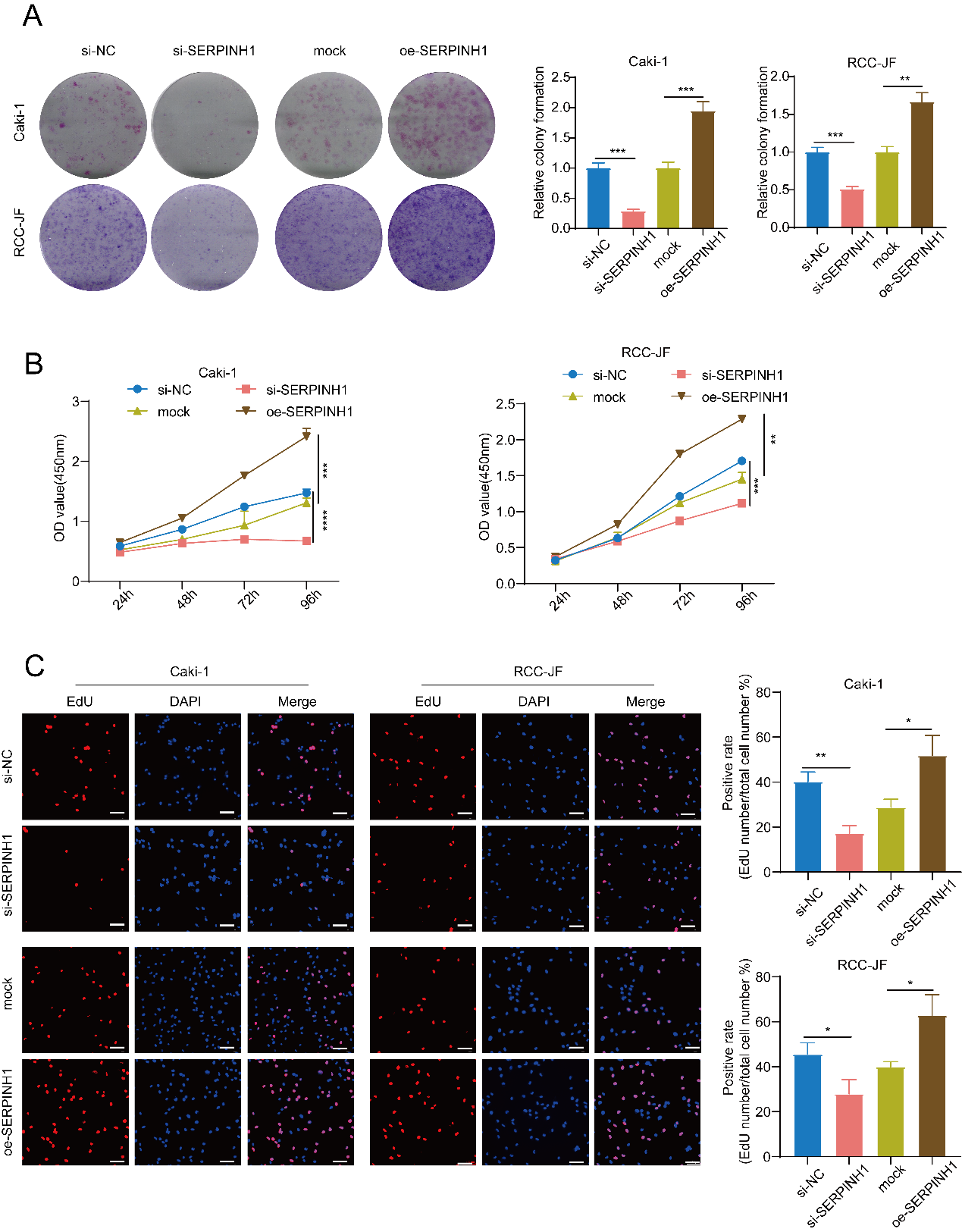


**Figure S11. SERPINH1 promotes the proliferation of ccRCC cells in vitro.** (A, B and C) Proliferation ability was examined by colony formation, CCK-8, and EdU assays after inhibition or overexpression of SERPINH1 in the Caki-1 and RCC-JF cells. Scale bar, 50 μm. Data were presented as mean ± SD. Student's t‐test, n = 3. * *P* < 0.05, ** *P* < 0.01, *** *P* < 0.001.


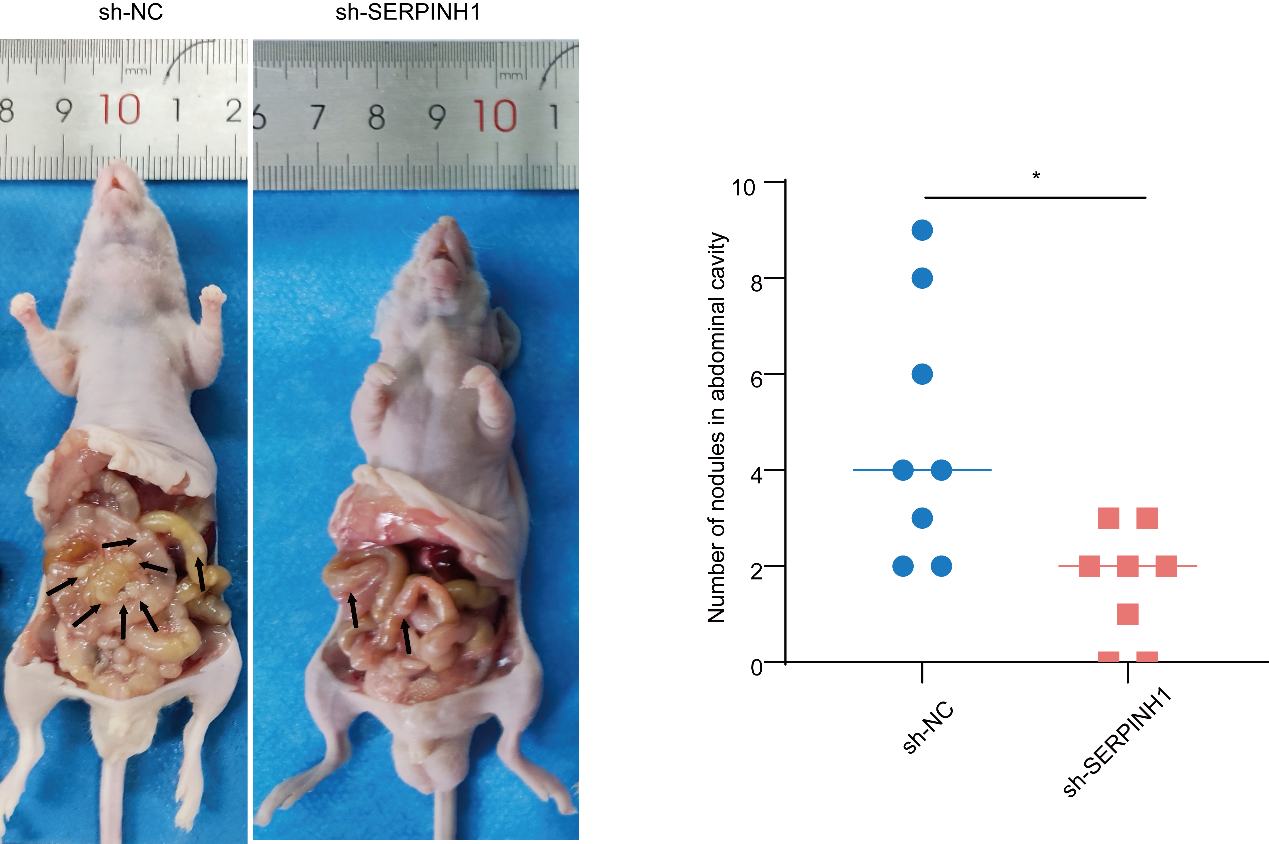


**Figure S12. SERPINH1 promotes metastasis of xenogeneic tumors in nude mice.** Representative images of the abdominal dissemination model (left) and the quantification of abdominal metastatic colonization (right). Mann-Whitney U test, n = 8. * *P* < 0.05.


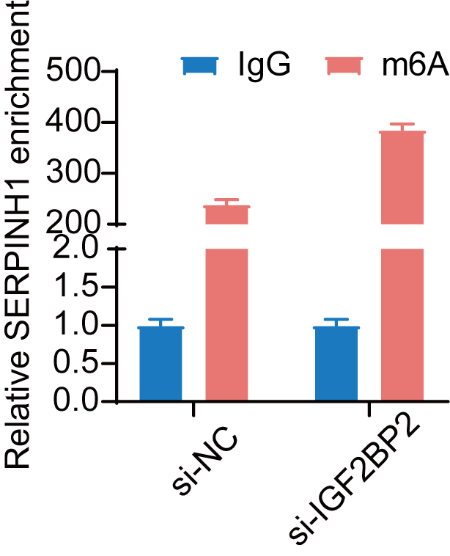


**Figure S13. IGF2BP2 may inhibit the expression of SERPINH1 by its m6A modification.** RIP experiments were performed with m6A antibody to verify the level of SERPINH1 m6A modification in Caki-1 cells interfering with IGF2BP2 or siRNA control.
